# The IL-33/ST2 axis and tissue Treg maintain epithelial homeostasis and restrain cancer development in the skin

**DOI:** 10.1016/j.celrep.2025.115837

**Published:** 2025-06-14

**Authors:** Sophie Ward, Greg Crawford, Buang Norzawani, Christina Malactou, Emma Dutton, Isabella Withnell, Kevin J. Woollard, Catherine Harwood, Henry J. McSorley, Christoph Ziegenhain, Adrian Lärkeryd, Anguraj Sadanandam, Jessica Strid

**Affiliations:** 1 Department of Immunology and Inflammation, Imperial College London, London, UK; 2 Centre for Cell Biology and Cutaneous Research, Queen Mary University of London, London, UK; 3 Division of Cell Signalling and Immunology, School of Life Sciences, University of Dundee, Dundee, UK; 4 Department of Medical Biochemistry and Biophysics, Karolinska Institutet, Stockholm, Sweden; 5 Centre for Translational Immunotherapy, The Institute of Cancer Research, London, UK; 6 Division of Molecular Pathology, The Institute of Cancer Research, London, UK

## Abstract

Interleukin (IL)-33 is constitutively expressed in many epithelial tissues at steady state and signals through the receptor, ST2. IL-33 is released upon tissue injury and functions as an endogenous danger signal to alert the immune system to tissue damage. Here we investigate the physiological role of the IL-33/ST2 axis in skin homeostasis and cancer development. We show that the expression of IL-33 differentiates malignant from normal and benign human tissues and that in mouse models of cutaneous squamous cell carcinoma the IL-33/ST2 axis protects against carcinogenesis. Tissue regulatory T cells (Tregs) are the predominant cells expressing ST2 in the skin and localize around the hair follicle and IL-33^+^ epithelial cells (ECs). Adoptive transfer experiments demonstrate that skin Tregs regulate EC differentiation, minimizing mutational load and restraining cancer development after exposure to an environmental carcinogen. Our findings indicate an important role for EC-Treg cross-talk as an early checkpoint for containing tissue damage and carcinogenesis.

## Graphical abstract

**Graphical abstract:**
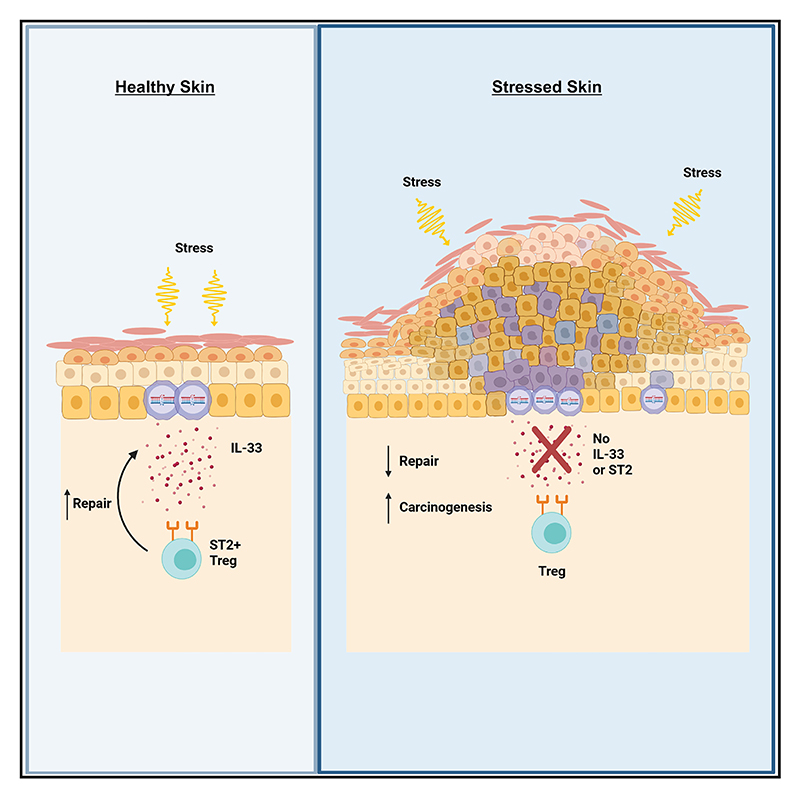

## Introduction

Interleukin-33 (IL-33) was first discovered in 2003 as a nuclear factor found in high endothelial venule endothelial cells^1^ and soon after, it was identified as a member of the IL-1 family that signals through its unique receptor ST2 (suppression of tumorigenicity 2, also known as IL-1RL1, T1, and IL-33R) in complex with the co-receptor IL-1RAcP.^2^ Since its discovery, IL-33 has emerged as a crucial cytokine in tissue homeostasis and in responses to environmental insults. IL-33 is constitutively expressed in the nuclei of a variety of cell types in steady state, particularly in epithelial cells (ECs) and endothelial cells and some fibroblasts.^3^ It is released into the extracellular space upon cell injury or tissue stress and functions as an endogenous danger signal to alert the immune system to tissue damage.

Many epithelial barrier tissues have a propensity to drive type 2 immunity upon non-invasive tissue damage and indeed the main targets for IL-33 are tissue-resident immune cells involved in type 2 immunity.^4^ As such, the bulk of work regarding IL-33 has revolved around its detrimental role in type 2 allergic diseases such as asthma and atopic dermatitis.

Dysregulated IL-33/ST2 signaling has also been described to play a pivotal role in a number of inflammatory, autoimmune, and metabolic disorders, including cardiovascular disease, inflammatory bowel disease, rheumatoid arthritis, systemic sclerosis, and obesity.^3^ In the skin, EC-derived IL-33 has been described to have both protective and immunopathological roles. For example, EC-specific over-expression of IL-33 induces a spontaneous dermatitis-like disease in mouse skin^5^ and IL-33 has been reported to be upregulated in human atopic dermatitis and psoriasis, as well as in the skin and serum of people with sclerosis.^6,7^ Conversely, treatment of mice with exogenous IL-33 produces dramatically improved wound healing, collagen deposition, and expression of extracellular matrix proteins in response to full-thickness skin wounding, indicating more efficient tissue repair.^8^ Together, these reports suggest that immediate production of IL-33 may be important in cutaneous homeostasis and integrity, whereas a constitutive, late, or dysregulated expression may be involved in a variety of chronic inflammatory conditions. This temporally coordinated function of IL-33 is consistent with the idea that it acts as an alarmin, whereby its immediate release from intracellular stores in damaged cells, facilitates a rapid and restorative response to tissue damage.

Lymphoid stress surveillance by γδ T cell receptor (TCR)^+^ intraepithelial lymphocytes (IELs) in the skin is key to the canonical stress response and the ability of these epidermal IELs to detect EC stress, caused by endogenous or exogenous insults and initiate a restorative response, play a pivotal role in regulating tissue homeostasis and tissue integrity.^9–12^ In addition, our previous data have shown that the ability of IELs to detect dysregulated ECs, pre-malignancy, is important for host protection against carcinogenesis.^9^ Indeed, this work has also revealed a critical host-defensive role for type 2 immunity in regulating EC tissue homeostasis and carcinogenesis.^11,12^ Although skin IELs can regulate the expression of IL-33 in adjacent ECs,^10,11^ it remains unclear how IL-33 contributes to the lymphoid stress surveillance response in the skin. Recent studies suggest a pleiotropic role for IL-33 in cancer immunity, with both pro- and anti-cancer effects of IL-33 having been reported.^13,14^ Given the variety of immune cell targets of IL-33, the multifaceted effect of the IL-33/ST2 axis in relation to cancer may thus depend on the temporal expression of IL-33, the tumor type, and cooperating microenvironmental factors.

Here we explore the role of the IL-33/ST2 axis in skin homeostasis and cancer development. We show that IL-33 is strongly downregulated in a variety of human cancers and that high IL-33 expression correlates with prolonged patient survival. In mouse models, we demonstrate that IL-33 is released from skin ECs after exposure to environmental carcinogens and that the presence of IL-33 and ST2 regulates EC differentiation and protects against cutaneous squamous cell carcinoma (cSCC) development. In addition, we demonstrate that skin resident regulatory T cells (Tregs) are the main expressors of ST2 in the skin and that skin Tregs reduce epidermal hyperplasia and promote epithelial development and integrity following exposure to carcinogenic xenobiotics. Ultimately, the presence of Treg in the tissue during carcinogen exposure reduce the mutational burden and restrains cancer development. Overall, our data suggests that direct EC-Treg cross-talk via the IL-33/ST2 axis in the skin promotes epithelial homeostasis and limits carcinogenesis, underlining the importance of an integrated local tissue response to acute environmental challenges.

## Results

### The IL-33/ST2 axis associates with better outcomes in several human cancers

**Figure 1:**
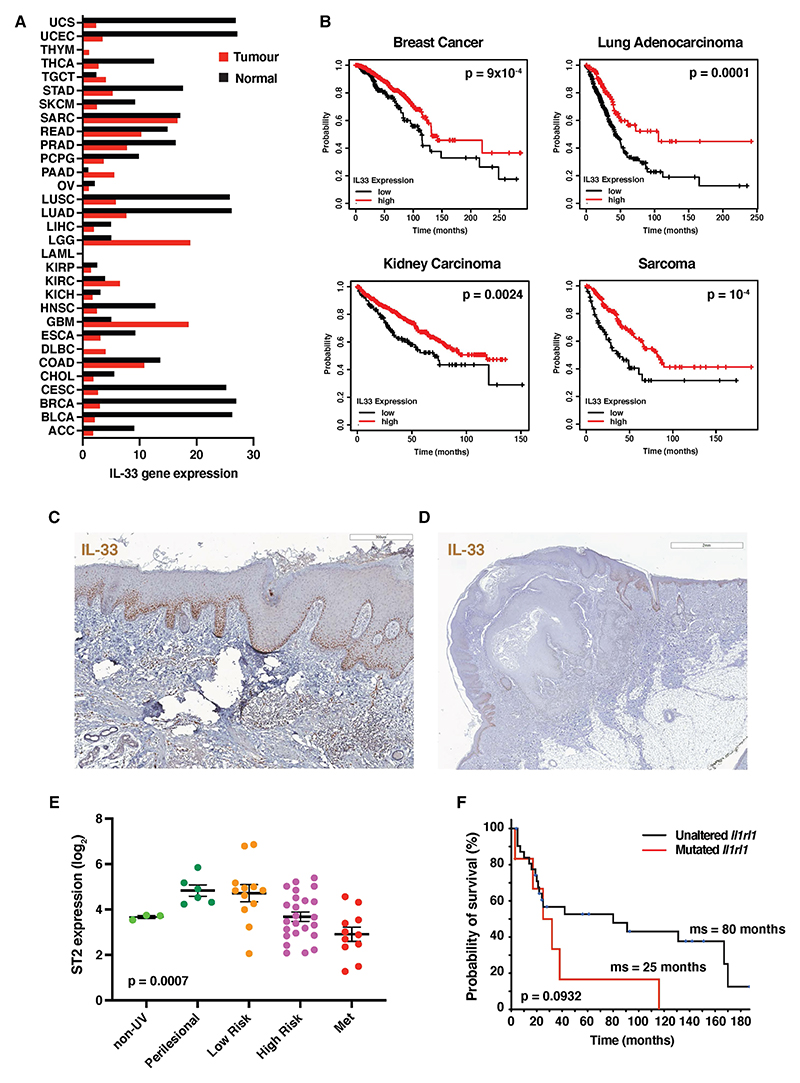
The IL-33/ST2 axis associates with better outcome in several human cancers (A) IL-33 gene expression profile in paired tumor samples and normal tissues. The height of the bar represents the median *Il33* expression as calculated by GEPIA.^15^ A key to the tumor abbreviations is included in the methods section. (B) Kaplan-Meier plots depicting the correlation between the expression of *Il33* and patient survival in selected cancers. Red line (above median *Il33*), black line (below median *Il33*). Analysis by KM-Plotter.^15^ (C and D) Examples of Immunohistochemical staining of IL-33 protein in human FFPE tissue (C) showing perilesional skin and (D) cSCC. (E) Tissue transcripts of *Il1rl1* (encoding ST2) in whole human skin and cSCC tissue analyzed by NanoString nCounter. Tissue was collected and scored independently for tumor risk by an experienced dermatopathologist; non-UV (*n* = 3), peri-lesional (*n* = 6), low-risk cSCC (*n* = 12), high-risk cSCC (*n* = 24), and metastasis (*n* = 11). Log2 RNA counts of *Il1rl1* are shown for individual risk groups and presented as mean ± SEM. (F) Kaplan-Meier plot showing survival for patients with aggressive cSCC correlated with missense mutations in *Il1rl1* (red line) or with non-mutated *Il1rl1* (black line) as estimated by whole-exome sequencing (*n* = 39). Ms, median survival. Analysis and data from cBioportal.^15,16^ Statistics by Log rank test in (B and F) and by one-way ANOVA and testing for linear trend of expression between risk groups in (E).

Many tissues, especially epithelial tissues, have a high steady-state expression of IL-33. However, using the GEPIA tool,^15^ we found that IL-33 was downregulated in most human cancer tissue compared with equivalent normal tissue, especially in carci-nomas (Figure 1A). Furthermore, an above median expression of IL-33 correlated with significantly prolonged patient survival in several epithelial cancers, as well as in sarcoma (Figure 1B).^16^ In human cSCC, IL-33 protein was strongly expressed in basal ECs in the normal/hyperplastic skin at the edges of the tumor (Figures 1C and 1D), but was absent from the tumor mass itself (Figure 1D). In a cohort of 56 patients with cSCC, gene expression analysis revealed that expression of *Il1rl1* mRNA (encoding ST2) was greatest in unaffected skin areas and in low-risk tumors and lowest in high-risk and metastatic cSCC (Figure 1E). A significant linear trend was observed between the expression of *Il1rl1* mRNA and the risk of more advanced cSCC (Figure 1E). Next, using the cBioportal webserver, whole-exome sequencing of 39 aggressive cSCC cases was analyzed,^17,18^ revealing lower median survival rates in patients with missense mutations in *Il1rl1* (ST2) compared with those without *Il1rl1* mutations (Figure 1F). Although this did not attain statistical significance in this small patient cohort (*p* = 0.0932), more than 15% of patients with aggressive cSCC had mutations in *Il1rl1*, and these patients had a median survival time of 25 months compared with 80 months for patients without *Il1rl1* mutations. Together, these data suggest that local expression of IL-33 may be a marker that differentiates malignant from normal/benign human tissues and implicate the IL-33/ST2 axis in tumor immune regulation.

### IL-33 Is Constitutively Highly Expressed in Mouse Skin But is Reduced Upon Tissue Damage

**Figure 2:**
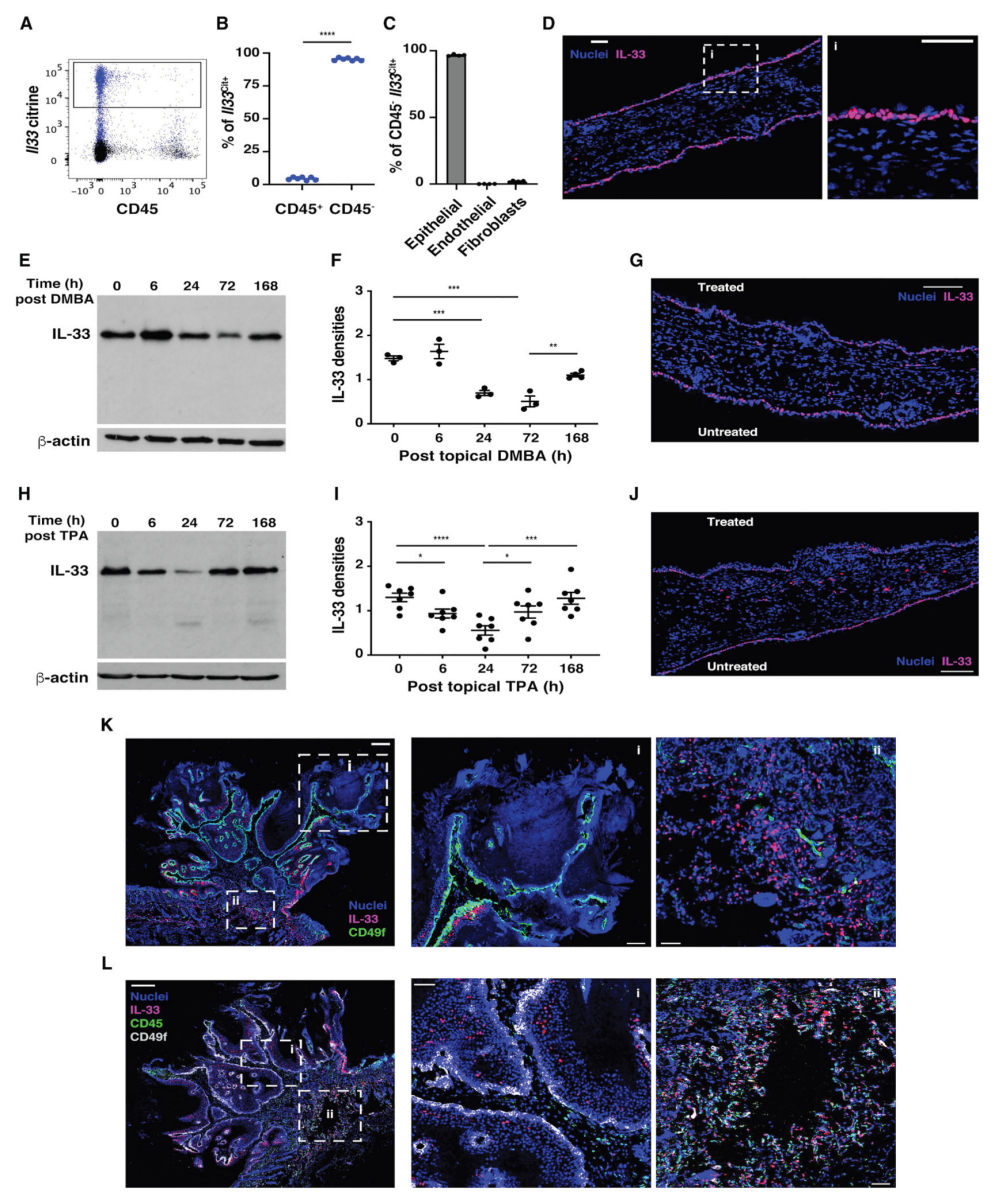
IL-33 is constitutively highly expressed in mouse skin but is reduced upon tissue damage (A) IL-33 expression in skin analyzed in (A) by flow cytometric analysis of skin digests from naive UT *IL33*^*Cit/Cit*^ reporter mice. *Il33*^*Cit+*^ in blue and *Il33*^*Cit*−^ shown in black. (B and C) (B) % *Il33Cit*^+^ cells that were CD45^+^ and CD45^−^ (*n* = 7) and (C) % *Il33Cit*^+^ CD45^−^ cells that were epithelial (EpCAM^+^), endothelial (CD31^+^CD105^+^), and fibroblasts (CD140a^+^). (D) Representative tile-scan of a cross-section of healthy UT ear skin stained for IL-33 protein (red) and nuclei (blue) with (di) showing magnification of indicated area. (E and H) Western blot analysis of IL-33 protein expression in epidermal lysates of shaved back skin from WT mice at indicated time points following a single topical exposure to (E) DMBA (*n* = 3 per time point) or (H) TPA (*n* = 7 per time point). (F and I) Abundance of IL-33 in the skin tissue blots expressed relative to b-actin abundance and normalized against an internal control sample. (G and J) Representative images showing IL-33 expression (red) in WT ear skin 72 h after topical exposure to (g) DMBA or (j) TPA. Nuclei in blue. (G and J) Representative images showing IL-33 expression (red) in WT ear skin 72 h after topical exposure to (g) DMBA or (j) TPA. Nuclei in blue. (K and L) Representative tile-scan images of WT mice with early stage cSCC (papilloma) indued by DMBA-TPA carcinogenesis showing (K) IL-33 (red), basement membrane (CD49f - gray), nuclei (blue) and (L) IL-33 (red), CD49f (gray), nuclei (blue), and CD45 (green). (i and ii) are showing magnifications of indicated areas. Scale bars in (D, G, and J) = 100 μm, (K and L) = 400 μm with (i, ii) = 100 μm. Imaging by confocal microscopy using ×63 tile scan (D, G, and J) and ×20 tile scan (K and L) and all shown images are representative of images from 4 to 10 individual mice. Data in (A–C, E, F, H, and I) are representative of two independent experiments with similar results and expressed as mean ± SEM (A, B, F, and I). Statistics by two-tailed Student’s *t* test for paired data (B) or one-way ANOVA for multiple comparison (F and I); *p < 0.05, **p < 0.01, ***p < 0.001 and ****p < 0.0001.

To directly investigate the impact of the IL-33/ST2 axis in skin dysregulation and carcinogenesis, we turned to mouse models. Using first an *Il33* citrine reporter mouse and flow cytometric analysis of single-cell skin suspensions, we found that IL-33 was constitutively and highly expressed in mouse skin, almost exclusively in CD45^−^ ECs (Figures 2A–2C). Immunofluorescence staining of steady-state skin from wild-type (WT) mice showed that IL-33 protein was localized to the nuclei of basal epidermal ECs (Figure 2D). Next, we investigated IL-33 expression during the early skin response to an environmental insult by exposing the skin to a single topical dose of the carcinogen 7,12-dimethyl-benz[*a*]anthracene (DMBA) or to the inflammatory agent 12-*O*-tetradeca-noylphorbol-13-acetate (TPA). DNA-damaging xenobiotics such as DMBA are commonly found in the environment and have been linked to carcinogenesis,^19,20^ whereas TPA causes epidermal hyperplasia and inflammation, hallmarks of a range of skin conditions.^21^ Western blotting (Figure 2E) and immunofluorescence (Figure 2G) showed that application of DMBA to the skin of WT mice caused a significant loss of IL-33 protein by 24 h, and this had only partly recovered by 168 h. A very similar significant reduction in nuclear epithelial IL-33 expression was seen following topical exposure to TPA, with slightly faster kinetics of recovery (Figures 2H–2J). Next, we assessed IL-33 expression in mouse cSCC, initiated by the widely used two-stage DMBA-TPA model.^22^ In early-stage cSCC (skin papillomas), we found IL-33 expression restricted to the less disrupted parts of the epithelium, whereas other parts of the tumors were devoid of IL-33 (Figure 2K). Instead, IL-33 was expressed now abundantly in the dermis directly underlying the tumor (Figures 2K and 2L), and this was predominantly in dermal CD45^−^ stromal cells (Figure 2L). Thus, epithelial dysregulation caused by topical exposure to environmental chemicals reduces epithelial IL-33 both acutely and in subsequent epithelial tumors.

### Tregs are the predominant expressors of ST2 in skin and tumors and localize in proximity to IL-33^+^ ECs

**Figure 3:**
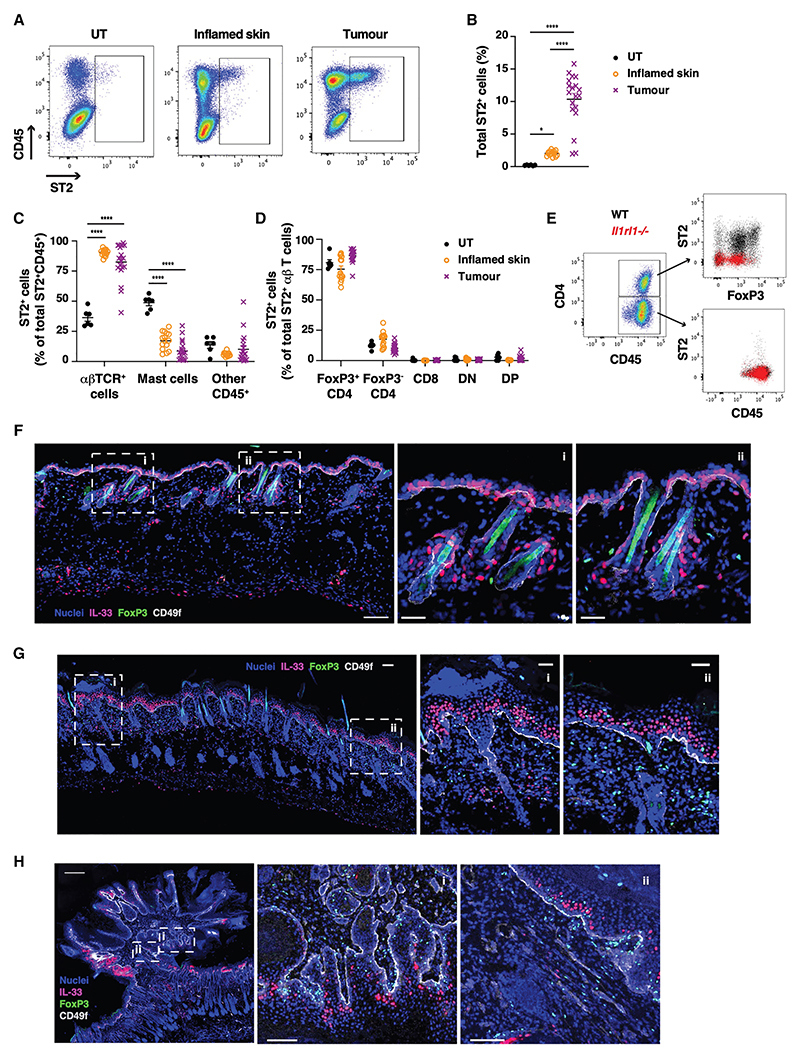
Tregs are the predominant expressors of ST2 in skin and tumors and localize in proximity to IL-33^+^ ECs (A–E) Flow cytometric analysis of ST2 expression on skin leukocyte populations from UT WT mice (*n* = 6), inflamed skin (induced by topical TPA twice a week for 3 weeks) (*n* = 14) or cSCC (induced by DMBA-TPA carcinogenesis and analyzed at 18 weeks) (*n* = 19). (A) representative dotplots showing CD45 and ST2 staining on whole tissue single cell suspensions and (B) the proportion of live cells expressing ST2. Data presented as mean ± SEM and statistics by one-way ANOVA multiple comparison; *p < 0.05 and ****p < 0.0001. (C) ST2 expression on αβ T cells (CD45^+^CD3^+^Tcrβ^+^), mast cells (CD45^+^cKit^+^FcεRI^+^) and other CD45^+^ leu-kocytes shown as proportions of all CD45^+^ST2^+^ cells. (D) ST2 expression by αβ T cell subsets as a proportion of all ST2^+^ αβ T cells. (E) Representative dotplots showing ST2 expression on tumor infiltrating CD4^+^ and CD4^−^ αβ T cells in WT mice (black) with cells from ST2-deficient (*Il1rl1*^−/−^) mice (red) shown as negative controls. (F and G) Representative tile scan images of skin tissue cross sections from WT mice (F) UT healthy skin, (G) inflamed skin (topical TPA twice a week for 3 weeks), and (H) cSCC (DMBA-TPA carcinogenesis) stained for IL-33 (red), FoxP3 (green), CD49f (gray) and nuclei (blue). (i, ii) are magnifications of indicated areas in (F–H). Imaging by confocal microscopy using ×63 tile scan (F) and ×20 tile scan (G and H). Images are representative of analysis of sections from 4 to 6 individual mice. Scale bars in (F and G) = 100 μm with (i, ii) = 50 μm, (H) = 400 μm with (i, ii) = 100 μm.

To explore which cells may respond locally to IL-33 in the skin, we examined expression of ST2 in untreated (UT) skin, in inflamed skin (induced by topical TPA), and in cSCC tissue (tumors induced by DMBA-TPA inflammation-driven carcinogenesis). We found that ST2 was expressed exclusively by CD45^+^ cells and that the relative abundance of cells expressing ST2 was significantly increased in dysregulated tissue compared with UT skin (Figures 3A and 3B). Although a mean of 0.2% of CD45^+^ cells expressed ST2 in UT skin, this increased to 2.0% in inflamed skin and to 10.4% in tumor tissue (Figures 3A and 3B). The cells in UT skin expressing ST2 were predominantly mast cells and αβTCR^+^ T cells with roughly equal distribution, but in inflamed skin and in tumor tissue, more than 85% of ST2^+^ cells were αβTCR^+^ T cells, with less than 10% mast cells. In all tissue conditions, few cells, other than mast cells and αβTCR^+^ T cells expressed ST2 (Figures 3C and S1). The vast majority of the αβTCR^+^ T cells expressing ST2 in all conditions were CD4^+^FoxP3^+^ Treg (>75%), with ST2 being found on only a small proportion of CD4^+^FoxP3^−^ cells and CD4^+^CD8^+^ double-positive cells (Figures 3D and 3E). A population of tissue-resident Treg has been reported previously in the skin, where they reside predominately around the hair follicle.^23^ Consistent with this, intravital microscopy showed that FoxP3^+^ cells were abundant in the skin of UT FoxP3^egfp^ reporter mice and these cells displayed a slow migration/scanning behavior, predominately around hair follicles (Figures S2a, S2b, and S2d–S2g). Conspicuously, ECs in steady-state hair follicles strongly expressed IL-33, as did basal ECs in other parts of the epidermis (Figure 3F). In inflamed skin, FoxP3^+^ cells increased in number, with more of these cells expressing lower levels of FoxP3 (egfp^lo^), as well as having enhanced motility and being more evenly distributed throughout the tissue (Figures S2c–S2g). Analysis of tissue cross-sections using immunofluorescence confirmed that inflamed skin harbored a large number of FoxP3^+^ cells in the dermis, as well as some on the other side of the basement membrane localized in close proximity to IL-33^+^ ECs (Figure 3G). FoxP3^+^ cells were also abundant in the peri-tumoral infiltrate in the cSCC model and were additionally observed in the tumor epithelium near IL-33^+^ ECs (Figure 3H). Expression of IL-33, or ST2, however, did not affect the accumulation or localization of Tregs in the tissue (Figure S3). Together, these data show that ST2 is predominately expressed on FoxP3^+^ Tregs in dysregulated skin and that these tissue Tregs localize close to IL-33^+^ ECs in the hair follicle and in the interfollicular epithelium.

### Topical carcinogen exposure promotes recruitment and accumulation of skin Tregs with a unique phenotype associated with tissue repair

**Figure 4:**
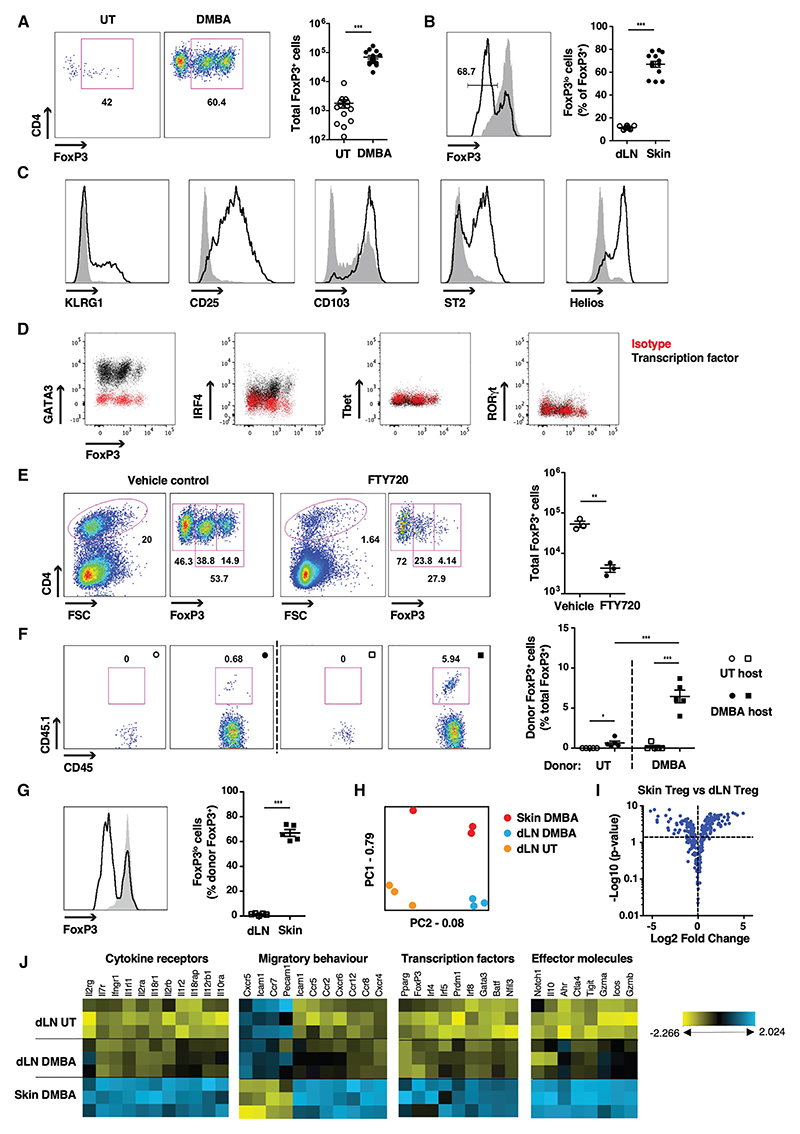
Topical carcinogen exposure promotes recruitment and accumulation of skin Tregs with a unique phenotype associated with tissue repair The dorsal ear skin of WT mice was topically exposed to the carcinogen DMBA twice, 3 days apart and the skin, skin dLNs and spleen were analyzed 7 days later by flow cytometry (A–G) or NanoString (H–J). (A) Representative dotplots showing CD4^+^FoxP3^+^ T cells in the skin of UT or DMBA-treated mice and graph depicting total numbers of FoxP3^+^ skin Tregs (UT *n* = 14; DMBA *n* = 17). (B) Representative histogram showing FoxP3 levels (MFI) in skin (black line) versus dLN (gray fill) Tregs and graph demonstrating the relative proportion of FoxP3^lo^ Tregs (as % of total FoxP3^+^ cells) in dLN and skin (*n* = 16). (C) Typical histograms comparing CD4^+^FoxP3^+^ Treg (black line) and CD4^+^FoxP3^−^ effector T cells (gray fill) in the skin. (D) Representative dotplots showing expression of FoxP3 and key transcription factors (black dots) in skin CD4^+^ T cells. Isotype control staining in red dots. (E) The sphingospine-1-phosphate inhibitor FTY720 was injected i.p. at 3 mg/kg 24 h before second dose of DMBA and then daily until the end of experiment. Dotplots show typical proportions of CD4^+^ and FoxP3^+^ Tregs in the skin and the graph demonstrates the total number of CD4^+^FoxP3^+^ Tregs after FTY720 or vehicle control treatment (*n* = 3). (F) Lymphocytes from UT or DMBA-treated CD45.1 donor mice were adoptively transferred to CD45.2 host mice, which were then left UT (open symbols) or DMBA treated (filled symbols. Dotplots depicts total donor FoxP3^+^ T cells (CD45.1^+^) in the skin of a typical host with the graph showing the proportion of donor FoxP3^+^ Treg as a % of total skin Treg (*n* = 5). The histogram in (G) illustrates the level of FoxP3 expression (MFI) in donor skin Tregs (black line) versus donor dLN Treg (gray fill) with the graph demonstrating the proportion of FoxP3^lo^ donor Treg in the skin versus dLN (*n* = 5). (H–J) Gene expression analysis of Tregs from dLNs and skin of UT and DMBA-treated mice using the NanoString nCounter gene expression panel NS_Immunology_Mm_C2269. (H) PCA of gene transcripts in Treg from skin (red) and dLN (blue) of DMBA-exposed mice and from UT dLN (orange). (I) Volcano plot illustrating differentially expressed genes in skin versus dLN Treg following topical DMBA exposure. Horizontal dotted line represents the threshold for statistical significance, *p* < 0.05. (J) Heatmap analysis indicating relative expression levels of genes of interest grouped as cytokine receptors, migratory behavior, transcription factors, and effector molecules. Each symbol (A, B, E, and F) represents an individual mouse with horizontal bars indicating mean ± SEM. Statistics by two-tailed unpaired Student’s *t* test (A, B, E, and F); *p < 0.05, **p < 0.01 and ***p < 0.001. (A–G) Data are representative of three independent experiments with similar results.

Since ST2 was strongly expressed mainly on Treg in dysregulated skin, we next examined the regulation and phenotype of tissue Treg after exposure to an environmental carcinogen. We found that DMBA exposure led to a marked increase in both the percentage and total number of CD4^+^FoxP3^+^ T cells in the skin (Figure 4A). Of note, FoxP3 expression in skin Treg was not uniform but displayed a clear bimodal pattern, with approximately 70% of Tregs possessing a FoxP3^lo^ phenotype (Figures 4A and 4B). This was in clear contrast with Tregs from the skin draining lymph node (dLN), which uniformly were FoxP3^hi^ (Figure 4B). However, skin Tregs still maintained features of conventional Treg such as IL-10 production; indeed, a significantly greater proportion of skin Tregs produced IL-10 *ex vivo* compared with dLNs Treg (Figures S4a and S4b). Although there was no difference in transforming growth factor b1 expression, we further found that skin Treg displayed high levels of the tissue repair factor, amphiregulin (AREG) (Figure S4b), which has been associated with the capacity of Tregs to promote tissue repair.^24^ *In situ* FoxP3^+^ skin Treg expressed higher levels of tissue residency and activation markers, relative to Foxp3^−^ effector CD4 T cells, including CD25, CD103, and KLRG1, as well as more ST2 (Figure 4C). The expression of all these markers was equivalent in Foxp3^hi^ and Foxp3^lo^ skin Treg (data not shown). In addition, FoxP3^+^ skin Tregs expressed high levels of the transcription factor Helios, commonly used as a marker for thymic-derived natural Tregs (Figure 4C), suggesting that the FoxP3^lo^ cells were not inducible Treg derived from FoxP3^−^ cells. Topical exposure to noxious environmental xenobiotics drives potent type 2 immunity in the skin,^12^ and it has been argued that tissue Tregs exhibit functional specialization to regulate tissue-specific immunity.^25^ Indeed, we found that skin Treg expressed high levels of the type 2 lineage-specific transcriptions factors GATA3 and IRF4, but not Tbet or RORγt (Figure 4D).

To examine the source of the skin tissue Treg response, we first assessed whether Treg activation occurred in the dLNs following skin damage by topical DMBA exposure. This showed a significant increase in both the percentage and total number of Tregs in the dLNs, which appeared to be due to proliferation as shown by a 2-fold increase in Ki67 staining of CD4^+^Foxp3^+^ T cells (Figures S5a and S5b). In contrast with the skin, Foxp3 expression levels remained uniformly high in the dLNs and, indeed, this increased further in response to DMBA (Figure S5c). dLN Tregs also showed increased expression of CD25, KLRG1, CD103, and ST2 after topical DMBA (Figure S5d and S5e), indicative of Treg activation. To further delineate the changes in dLN Tregs during this priming process, Nanostring analysis was carried out on dLN Tregs from UT and topical DMBA treated animals. Forty-seven genes were differentially expressed after DMBA-induced skin dysregulation, including transcription factors such as *Batf, Nfil3*, and *Gata3*, chemokine receptors including *Ccr2* and *Cxcr6*, soluble mediators *Gzma* and *Gzmb* (encoding granzymes A and B), and stress-sensing receptors including *Ahr* and *Il1rl1* (genes highlighted) (Figure S5f). These data suggest that Tregs primed in skin dLNs are specialized for tissue effector function and may contribute to the skin tissue Treg response. To investigate directly whether skin Tregs were recruited from dLNs after DMBA-induced skin damage, we carried out lymph node egress blocking experiments, using the sphingospine-1-phosphate inhibitor, FTY720. Administration of FTY720 during topical DMBA exposure led to a 10-fold decrease in the numbers of Foxp3^+^ cells in the skin (Figure 4E), supporting the idea that these cells are indeed recruited from the dLNs Tregs.

To understand the dynamics of the tissue Treg response better, we next tracked the ability of naive or DMBA-primed lymphocytes from dLNs to give rise to Treg in healthy or DMBA-exposed skin. No donor-derived Tregs could be found in the skin after transfer of naive donor lymphocytes into naive hosts, and only low numbers of naive donor cells were recruited into the skin after topical DMBA exposure of the host, despite the expansion of endogenous Treg in these mice (Figure 4F). However, when the donor cells were taken from the dLNs of animals exposed to topical DMBA before transfer, substantial numbers of donor-derived Treg accumulated in DMBA-treated skin (Figure 4F). Interestingly, donor-derived skin Tregs had down-regulated FoxP3 levels after recruitment to the skin tissue (Figure 4G), suggesting that FoxP3 expression is regulated by local microenvironmental cues.

To further examine changes in Treg biology associated with epithelial dysregulation and tissue localization, we compared the transcriptome of dLNs and skin Tregs using the NS_Immunology_Mm_C2269 Nanostring panel. Principal component analysis (PCA) showed that DMBA-primed dLNs and skin Tregs clustered separately from each other, with 168 of the 547 genes in the panel being significantly differentially expressed (*p* < 0.05) (Figures 4H and 4I). These included cytokine receptors, chemokine receptors, and adhesion molecules associated with migration into non-lymphoid tissues, transcription factors, and effector molecules (Figure 4J), suggesting specific tissue adaptation. Tregs from UT and DMBA-primed dLNs also clustered separately, highlighting the activation of Treg in dLN after epithelial dysregulation (Figures 4H and S5f). Taken together, these data show that topical exposure of the skin to damaging xenobiotics primes Treg in the dLNs, which migrate to the skin and rein-force skin Treg numbers with a tissue-specific phenotype.

### ST2 expression defines an activated population of skin Tregs

**Figure 5:**
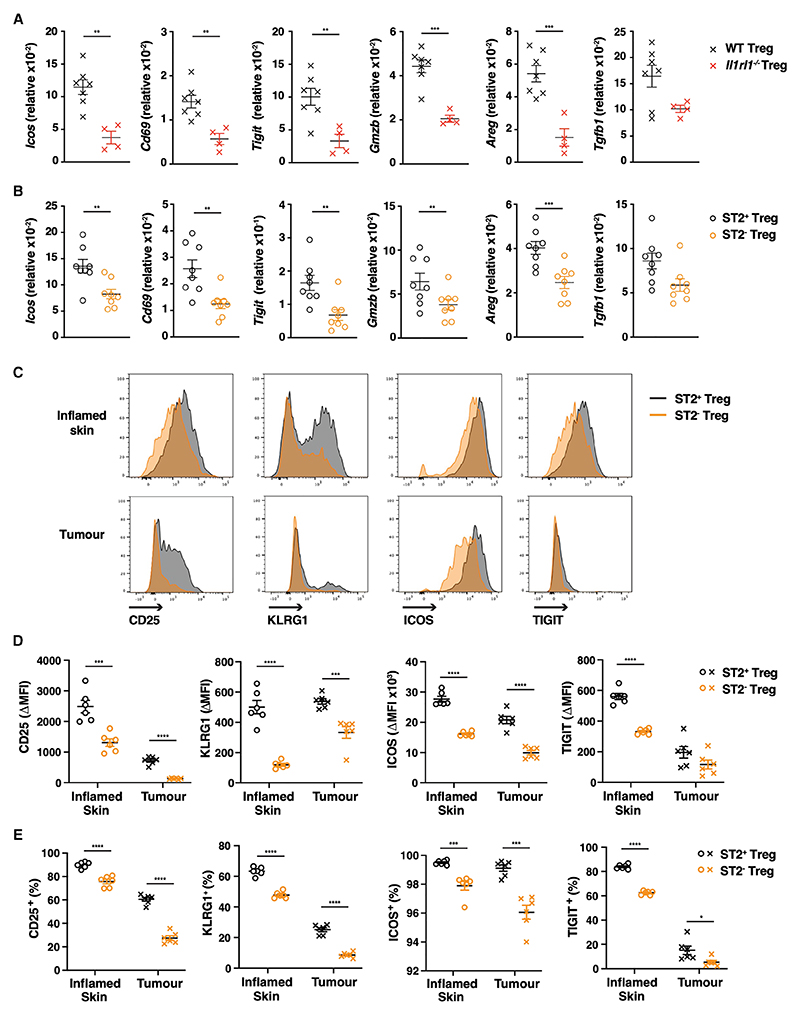
ST2 expression defines an activated population of skin Tregs (A and B) TCRβ^+^CD4^+^CD25^hi^CD103^+^ Treg were sorted by FACS from (A) cSCCs on WT (*n* = 7) and *Il1rl1*^−/−^ (*n* = 4) mice and (B) inflamed skin of WT mice (*n* = 8) additionally sorted into ST2^+^ and ST2^−^ Tregs. Tumors were induced by DMBA-TPA inflammation-driven carcinogenesis and collected at week 20. Skin inflammation was induced by topical exposure to TPA alone. Total RNA was extracted from Treg and gene expression of indicated genes were assessed by qRT-PCR and expressed relative to the housekeeping gene cyclophilin (*Cyc*) using 2^−ΔCt^. Data are representative of two independent experiments with similar results. (C) Representative histograms showing expression of indicated markers and quantification of (D) level of expression and (E) proportion of positive Treg on inflamed skin ST2^+^ Tregs and tumor ST2^+^ Tregs (black) versus ST2^−^ Tregs (orange) from within the same tissue. Inflamed skin and tumors were induced on WT mice (*n* = 6) as in (A and B) and tissue Tregs (TCRβ^+^CD4^+^CD25^+^FoxP3^+^) were analyzed by flow cytometry. Data are presented as mean ± SEM and statistics by unpaired *t* test; **p < 0.01, ***p < 0.001 and ****p < 0.0001.

To understand the effect of ST2 signaling on the tissue Treg phenotype, we next compared skin and tumor Treg from WT and ST2-deficient animals (*Il1rl1*^−/−^) as well as WT ST2^+^ and ST2^−^ Tregs from within the same tissue. We found that Treg fluorescence activated cell sorted (FACS) from cSCCs of WT animals expressed more *Icos, Cd69, Tigit, Gmzb*, and *Areg* than Treg sorted from tumors of *Il1rl1*^−/−^ mice (Figure 5A) (analogous results were obtained when Treg were sorted from tumors of *Il33*^−/−^ mice) (Figure S6). Similarly, ST2^+^ Tregs sorted from tumor-bearing skin of WT mice expressed higher levels of these genes than ST2^−^ Tregs from the same tissue (Figure 5B). Flow cytometric analysis of surface proteins confirmed that a greater proportion of ST2^+^ Tregs from both tumor tissue and inflamed skin expressed CD25, KLRG1, ICOS and TIGIT than ST2^−^ Tregs from the same tissues; the levels of these markers were also significantly higher on ST2^+^ Tregs in both inflamed skin and tumors (Figures 5C–5E). Thus ST2 expression marks Tregs with a phenotype indicative of activation and tissue protection, suggesting an important role for IL-33/ST2 signaling in skin Tregs.

### The IL-33/ST2 axis protects against cancer development and limits tumor EC dysregulation

**Figure 6:**
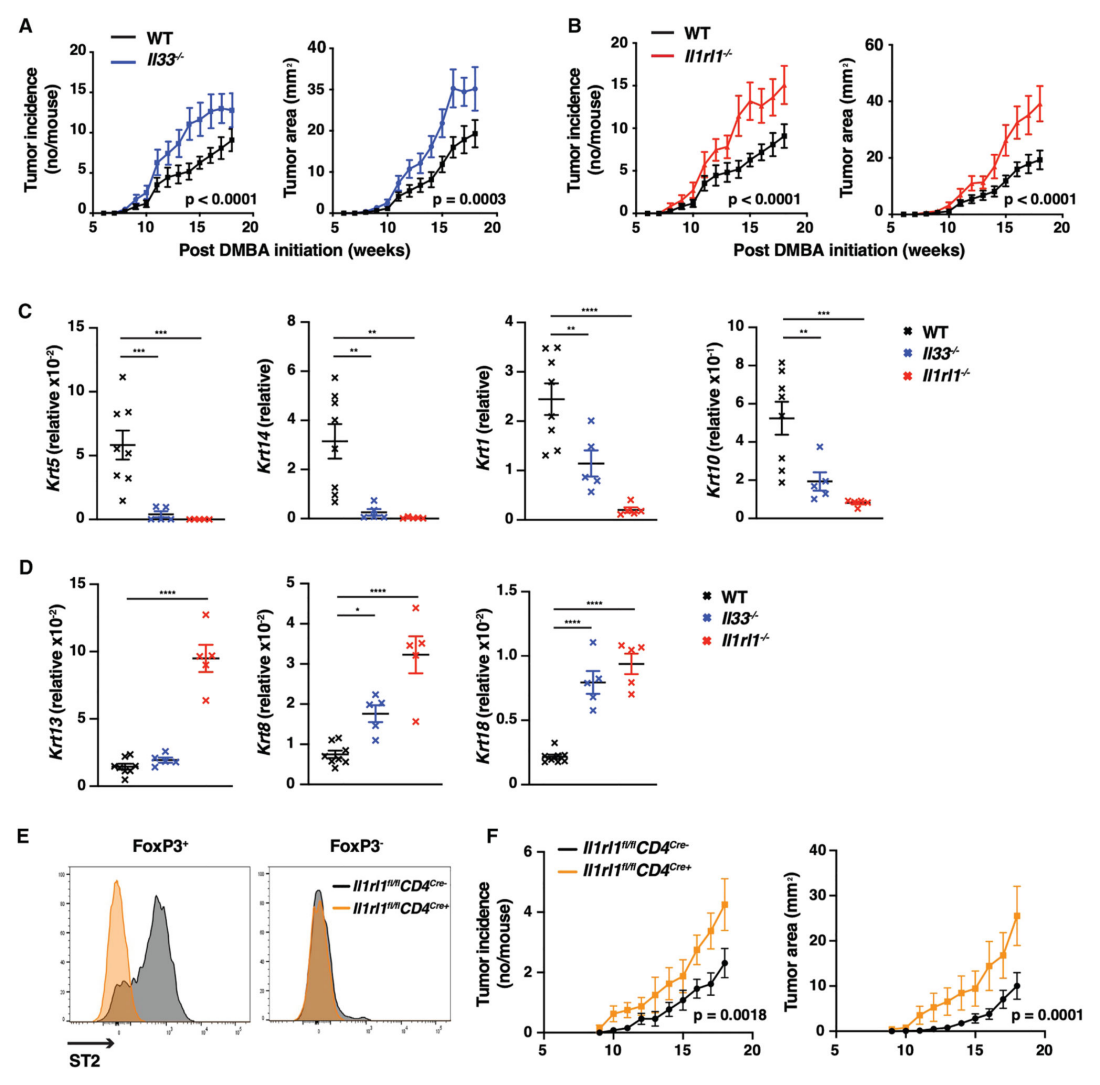
The IL-33/ST2 axis protects against cancer development and limits tumor EC dysregulation (A and B) cSCCs was induced by DMBA-TPA carcinogenesis on (A) WT (*n* = 11) and *Il33*^−/−^ (*n* = 14) mice, (B) WT (*n* = 11) and *Il1rl1*^−/−^ (*n* = 11) mice, and (F) *Il1rl1*^*fl/fl*^ *CD4*^*Cre-*^ (*n* = 13) and *Il1rl1*^*fl/fl*^*CD4*^*Cre+*^ (*n* = 8) mice. Tumor susceptibility is expressed as tumor incidence (average number of tumors per mouse) and tumor area (average tumor size per mouse). Data are representative of two independent experiments with similar results and expressed as means ± SEM. Statistical significance assessed using linear regression. (C and D) Tumors from (A and B) were collected at 20 weeks and CD45^−^ ECs were sorted by FACS and total RNA collected (WT [*n* = 8], *Il33*^−/−^ and *Il1rl1*^−/−^ [*n* = 5]). qRT-PCR analysis of keratin genes associated with (C) EC differentiation and (D) malignant tumor conversion was performed and expressed relative to the housekeeping gene cyclophilin (*Cyc*) using 2^-ΔCt^. Statistics by one-way ANOVA multiple comparison; *p < 0.05, **p < 0.01, ***p < 0.001 and ****p < 0.0001. (E) Representative histograms showing expression of ST2 on CD4^+^FoxP3^+^ Treg and CD4^+^FoxP3^−^ effector T cells in the tumors from *Il1rl1*^*fl/fl*^*CD4*^*Cre*−^ littermate controls and *Il1rl1*^*fl/fl*^*CD4*^*Cre+*^mice.

To explore the functional impact of the IL-33/ST2 axis during EC dysregulation and cancer development, we analyzed the consequences of its absence during DMBA-TPA inflammation-driven carcinogenesis. We found that mice lacking IL-33 (*Il33*^−/−^) (Figure 6A) or ST2 (*Il1rl1*^−/−^) (Figure 6B) were significantly more susceptible to tumor development than their age-and sex-matched WT controls. Both IL-33- and ST2-deficient mice had an increased incidence of tumors as well as growing bigger tumors than their WT counterparts (Figures 6A and 6B). However, flow cytometry of the tumor immune infiltrate revealed no significant differences in the abundance of total leukocytes in WT or IL-33- or ST2-deficient tumors, nor was there any differences in the relative distribution of any of the major lymphoid or myeloid sub-populations, including FoxP3^+^ Treg (Figures S7a–S7d). qRT-PCR analysis of FACS-sorted CD45^−^ cells showed that, compared with WT tumors, those in IL-33- and ST2-deficient mice displayed strong downregulation of keratin genes involved in EC differentiation, including *Krt5* and *Krt14*, expressed by basal ECs, and *Krt1* and *Krt10* expressed by suprabasal ECs (Figure 6C), indicative of poorly differentiated tumors in the absence of the IL-33/ST2-axis. In addition, IL-33- and ST2-deficient tumor ECs showed significantly enhanced expression of *Krt13, Krt8*, and *Krt18* (Figure 6D), which are associated with malignant conversion in cSCC. To exclude a role for ST2 signaling on non-T cells, we further analyzed the tumor susceptibility of *Il1rl1*^*fl/fl*^*CD4*^*Cre+*^ mice. These mice did not have an altered abundance of tumor leukocytes but showed no expression of ST2 on Treg (or on FoxP3^−^ CD4 T cells, but these cells expressed very limited ST2 also in *Il1rl1*^*fl/fl*^*CD4*^*Cre*−^ littermate controls) (Figures 6E and S8). The conditional ST2-deficient mice also showed an enhanced susceptibility to tumor development, demonstrating an increased incidence and enlarged size of tumors (Figure 6F). Together these results demonstrate that absence of the IL-33/ST2 axis enhances skin cancer susceptibility and causes tumor EC dysregulation.

### Skin Tregs reduce epidermal hyperplasia and promote epithelial health after carcinogen exposure

**Figure 7:**
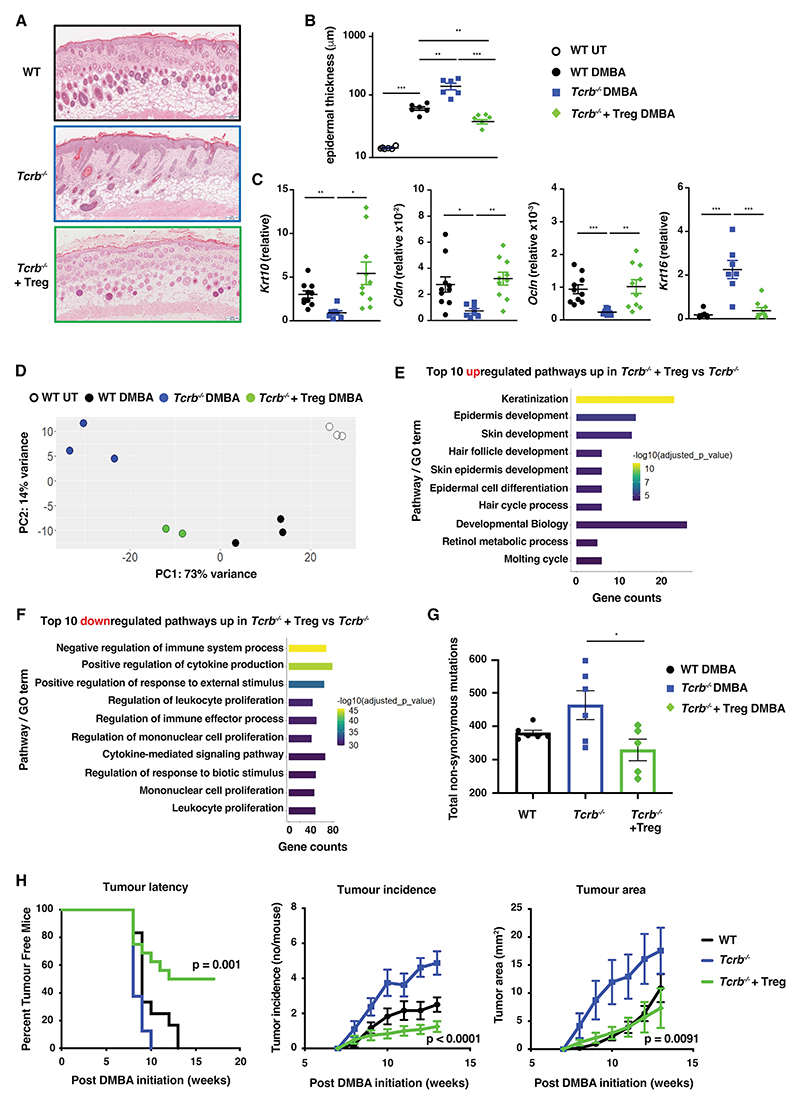
Skin Tregs reduce epidermal hyperplasia and skin mutational burden following carcinogen exposure and limit tumor development CD3^+^CD4^+^CD25^hi^ cells were purified from spleen and LNs of WT mice and 5 × 10^5^ cells were injected i.v. into αβ T cell-deficient mice (*Tcrb*^−/−^). At 24 h after adoptive Treg transfer, the back skin was exposed to the carcinogen DMBA and the treatment was repeated once weekly for a total of 6 weeks (A–G) or 12 weeks (H). Adoptively transferred *Tcrb*^−/−^ + Treg mice were compared with similarly DMBA-treated *Tcrb*^−/−^ and WT mice. (A) Representative images of hematoxylin and eosin-stained cross-sections of DMBA-treated back skin and (B) epidermal thickness enumerated (*n* = 6 per group). (C) Total RNA was extracted from DMBA-treated back skin and expression of indicated EC-specific genes were analyzed by qRT-PCR and presented relative to expression of the housekeeping gene cyclophilin (*Cyc*) using 2^-ΔCt^ (*n* = 6 per group). (D–F) Bulk RNA sequencing was performed on whole back skin biopsies. (D) Clustering of RNA transcripts between groups by PCA. (E and F) gprofiler pathway analysis showing the 10 most (E) upregulated genes and (F) downregulated genes in the skin of *Tcrb*^−/−^ + Treg versus *Tcrb*^−/−^ mice similarly exposed to topical carcinogen. (G) DNA was collected from skin biopsies and whole exome sequencing performed (*n* = 6 for WT and *Tcrb*^−/−^, *n* = 5 for *Tcrb*^−/−^ + Treg). Mean *de novo* total non-synonymous mutation ± SEM are shown after filtering variants from UT naive controls. (H) Tumor susceptibility is expressed as tumor latency (time to appearance of first tumor), tumor incidence (average number of tumors per mouse) and tumor area (average tumor size per mouse) in WT (*n* = 12), *Tcrb*^−/−^ (*n* = 9) and *Tcrb*^−/−^ + Treg (*n* = 12) mice. Data in (A–C) and (H) are representative of two independent experiments with similar results and expressed as mean ± SEM. Statistical significance assessed by one-way ANOVA multiple comparison (B, C, and G) and using a log-rank (Mantel Cox) test for tumor latency and linear regression for tumor incidence and area (H); stated significance in (H) delineates difference between the *Tcrb*^−/−^ and *Tcrb*^−/−^ + Treg group. *p < 0.05, **p < 0.01, ***p < 0.001.

To further understand the role of skin Tregs during early carcino-genesis, we first attempted to use the DEREG (depletion of Tregs) model in which transgenic mice express the diphtheria toxin receptor under the Foxp3 promoter (FoxP3^DTR^), allowing us to selectively deplete Tregs during the development of skin cancer (Figures S9a and S9b). The significant decrease in skin Tregs was associated with an enhanced infiltrate of CD4^+^ Foxp3^−^ effector T cells (Figure S9b). To study the consequences of loss of tissue Treg on skin cancer susceptibility, we used the DMBA-TPA carcinogenesis model and depleted Tregs only once weekly to minimize systemic effects. However, although the FoxP3^DTR^ mice remained healthy overall, they developed severe hyperplasia of the epidermis with scarring, intense inflammation, and signs of abnormal adipogenesis (Figure S9c), so experiments were halted before tumors became apparent. We then sought alternative models to investigate the ability of skin Tregs to directly impact epithelial health *in vivo* and set-up a T cell transfer model wherein purified CD3^+^CD4^+^CD25^hi^ Treg were transferred to αβ T cell-deficient mice (*Tcrb*^−/−^). These Tregs stably engrafted and, as in WT mice, increased in relative abundance (%) and in total number both in the skin dLN (Figures S10a and S10b) and in the skin (Figure S10c and S10d) following topical carcinogen exposure. Notably, the engrafted Treg also expressed significant levels of CD103 and ST2 following topical carcinogen exposure in dLNs, and even more so in the skin (Figure S10e). These mice, in the absence of autoreactive CD4^+^FoxP3^−^ effector T cells, tolerated topical carcinogen exposure well and we thus proceeded to compare the skin tissue of WT, *Tcrb*^−/−^, and *Tcrb*^−/−^ mice with engrafted Treg after repeated topical exposure to DMBA. At this premalignant stage, the epithelium is considerably dysregulated, but no clinically visible tumors are present. We found significant hyperplasia of the epithelium in WT mice, compared with UT WTs, and this was further increased in mice lacking all αβ T cells (*Tcrb*^−/−^) (Figures 7A and 7B). However, the introduction of skin Treg to *Tcrb*^−/−^ mice before carcinogen exposure significantly reduced the epidermal hyperplasia compared with *Tcrb*^−/−^, but also to WT mice (Figures 7A and 7B). In addition, the presence of Treg in *Tcrb*^−/−^ mice restored the expression of *Krt10* and the important tight junction genes *Cldn* and *Ocln* to WT levels, whereas the expression of inflammatory *Krt16* was reduced compared with *Tcrb*^−/−^ mice (Figure 7C), indicative of a less dysregulated EC response when Tregs were present in the tissue. Bulk RNA sequencing analysis and PCA of whole skin biopsies showed a clear difference in gene expression between UT WT and carcinogen-treated WT mice and also between WT and *Tcrb*^−/−^ mice exposed to carcinogens (Figure 7D). In addition, we found a distinct skin gene expression in *Tcrb*^−/−^ mice reconstituted with Tregs compared with similarly treated *Tcrb*^−/−^ mice (Figure 7D). Differential gene expression analysis showed that 936 genes were significantly differentially expressed (adjusted *p* < 0.01 and fold change of >2 or <−2) between carcinogen-treated *Tcrb*^−/−^ and *Tcrb*^−/−^ + Treg skin. Interestingly, pathway analysis using gprofiler revealed that the top 20 upregulated gene pathways in the skin after Treg reconstitution were all associated with skin EC development, keratinization, hair follicle development, and EC differentiation (top 10 pathways shown in Figure 7E and the genes in each pathway shown in Table S1) with important transcription factors for epidermal stem cell fate and epidermal development such as *Sox9* (*p* = 6 × 10^−8^), *Foxn1* (*p* = 10^−3^) and *Dlx3* (*p* = 10^−3^) highly differentially expressed in the presence of Treg. The top 20 downregulated pathways were related to inflammatory responses such as cytokine-signaling and leukocyte proliferation (top 10 pathways shown in Figure 7F and the genes in each pathway shown in Table S2). Furthermore, *in vitro* co-cultures showed that IL-33 endowed Treg with the ability to induce skin EC differentiation (Figure S11). Overall, these data demonstrate that skin Tregs can regulate EC differentiation and promote a healthier skin barrier after exposure to an environmental carcinogen.

### Skin Tregs reduce skin mutational burden after carcinogen exposure and limit tumor development

In light of the Treg-induced changes in the EC skin compartment and the apparent protection from carcinogen-induced damage by the presence of tissue Tregs, we next performed whole exome sequencing of the skin of WT, *Tcrb*^−/−^, and *Tcrb*^−/−^ mice with engrafted Tregs after repeated topical carcinogen exposure to explore the mutational landscape in premalignant tissue. All variants present in naive UT skin were filtered out. The vast majority of *de novo* variants after carcinogen exposure were missense mutations and these were increased in *Tcrb*^−/−^ skin over WT skin but significantly decreased in the skin of *Tcrb*^−/−^ mice reconstituted with Tregs before carcinogen exposure (Figure 7G). This suggests that the presence of Tregs is able to protect ECs from DNA damage or facilitate the expulsion of mutated cells from the skin.

Finally, to probe the functional role of Tregs during carcino-genesis and tumor development, we used the adoptive Treg transfer model before full carcinogen tumor (cSCC) induction. This demonstrated that, while all *Tcrb*^−/−^ mice had developed one or more tumors by 10 weeks, 50% of *Tcrb*^−/−^ mice reconstituted with Tregs were still tumor free at 17 weeks, when the experiment was terminated (Figure 7H), indicating a significantly prolonged latency in the presence of Tregs. Furthermore, *Tcrb*^−/−^ mice developed a significantly greater number of tumors that also grew larger than those in tumor-bearing *Tcrb*^−/−^ with Tregs or WT control mice (Figure 7H). These data strongly indicate that the presence of tissue Tregs at the time of cancer-initiating EC dysregulation protects against tumor development.

## Discussion

In this study, we found that IL-33 expression strongly related to the health status of the skin tissue and that IL-33 levels were reduced in malignancy. ST2, the receptor for IL-33, was expressed mainly on tissue Tregs, which showed tissue-specific adaptations, and were positioned close to IL-33^+^ ECs. We showed that the IL-33/ST2 axis protected against EC dysregulation and cancer development after exposure to carcinogenic xenobiotics. These effects reflected the ability of skin Treg to regulate EC differentiation, minimize epithelial dysregulation, and reduce mutational load after carcinogen exposure, resulting in decreased cancer susceptibility. We propose that tissue-resident Tregs promote epithelial differentiation and tissue homeostasis after an alert signal from damaged ECs via the alarmin IL-33. Ultimately, this strengthens epidermal defenses and limits EC cancer development.

That Tregs in this context protect against cancer development strongly contrast with the prevailing dogma that Tregs inhibit anti-tumor immune responses and promote cancer growth. However, it is now recognized that tissue Tregs differ in phenotype and function(s) from lymphoid organ Tregs and act as gatekeepers of tissue homeostasis.^25^ The first step to establish a tumor during tumor initiation is the disruption of tissue homeostasis.^26^ Thus, teleologically it seems reasonable that tissue Tregs may be limiting dysregulation during early carcinogenesis and, thus, restraining tumor initiation. As shown here, skin tissue Tregs promote EC differentiation and epidermal development and, as such, may limit the retention of damaged and/or mutated ECs in the tissue. The origin of malignancy involves the uncoupling of growth and differentiation and poor differentiation is a hallmark of cancer. Indeed differentiation therapy has been suggested as a promising strategy for cancer treatment.^27^ In addition, it is well established that chronic tissue inflammation can be a catalyst for tumor growth and progression, and Tregs may be beneficial in limiting tumor-promoting inflammation.

Clearly, context matters, and the situation may be different in established tumors. Indeed, the composition and the functional state of cells within the tumor microenvironment (TME) varies hugely not only with the tumor type but also with tumor stage and between individual patients. There is growing appreciation that organ-specific imprinting of cells under homeostatic conditions can explain differences in the function of cells within the TME of tumors in different tissues.^28^ However, non-tissue-specific immune cells also infiltrate tumors and with time cancer cells can orchestrate a tumor-supporting microenvironment. Many established solid tumors are heavily infiltrated by Tregs and a large body of literature suggests that an increased frequency of Tregs in the TME is associated with an adverse prognosis and that Treg depletion strategies in preclinical cancer models can induce effective anti-tumor immunity.^29^ We found that continued tissue damage and/or inflammation caused a large infiltration of lymphoid tissue Treg into the skin, which differed in their original transcriptome and in their tissue location and migration in the skin. Consistent with this, TCR repertoire analysis of Tregs in human cancer support the idea that intratumoral Tregs are unlikely to originate from expansion of tissue-resident Tregs; for example, the TCR repertoire of breast tumor Tregs shows little overlap with Tregs from normal matched breast tissue and in melanoma the TCR repertoires of intratumoral Tregs display significant overlap with peripheral blood Tregs.^30,31^ Nevertheless, our adoptive transfer model demonstrated that *de novo* tissue Tregs, established before tissue damage in the absence of endogenous Tregs, could also support EC differentiation and protect against cancer initiation. In established tumors, however, infiltrating lymphoid Tregs may not be able to receive tissue-specific signals to regain tissue homeostasis or may be subverted by the tumor to promote cancer cell growth.

We found that the expression of IL-33 is strongly reduced across most malignant tissues, and, in cSCC, that ST2 is significantly downregulated in high-risk and metastatic tumors compared with perilesional skin and low-risk tumors. Consistent with this, single-cell transcriptomics in melanoma have shown that ST2 is one of the most downregulated genes in tumor Tregs compared with healthy skin Tregs.^32^ Previous studies have found that IL-33 is an important mediator of the Treg tissue repair response; in a lung infection model of inflammation-induced tissue damage, Tregs were found to have a direct, non-redundant role in tissue repair in response to IL-33 and IL-18^24^ and ST2^+^ Tregs promoting tissue repair have also been reported in the gut and muscle tissue,^33,34^ as well as ST2^+^ Tregs promoting adipocyte differentiation and function.^35^ In healthy skin, we observed a strong expression of IL-33 proteins in the basal layer of the epidermis and in the hair follicles around the sebaceous gland and bulge area. In line with this, single-cell transcriptomics have shown that the proliferating EC population, marked by K14, indeed express IL-33, as does the stem cell population marked by the expression of Lgr6.^36^ These basal proliferating ECs and stem cell populations contribute to renewal and re-epithelialization after tissue damage.

Skin tissue Tregs seed the skin perinatally and localize in or near hair follicles in both mice^23^ and humans.^37^ Our data are consistent with this and shows a unique slow scanning behavior of the hair follicle Treg. These skin Treg have been shown to interact directly with hair follicle stem cells (HFSCs) to drive their proliferation and differentiation.^23^ It is likely that, during tissue damage, these Tregs respond to released IL-33 to promote tissue regeneration and epithelial differentiation. Two likely mechanisms are (1) via release of soluble EGFR ligands, for example AREG, which is enhanced by IL-33/ST2 signaling, and (2) via direct cell-cell contact involving Notch ligands and Notch receptor. It has been shown that Treg promotion of HFSC function in the skin is not due to suppression of inflammation but rather expression of Jagged1, which can signal to Notch-expressing HFSCs.^23^ These pathways are also known to control important transcriptions factors involved in epidermal development, differentiation, and stem cell fate, such as SOX9, FOXN1, and DLX3, which we found highly enhanced in the presence of skin Tregs. In cSCC, Notch is among the top frequently altered pathways, although contrary to many other cancer types, the Notch pathway has consistently been demonstrated to be tumor suppressive in cSCC.^38^ Equally, FOXN1 might be a necessary cue for the proper function of stem cells in the skin and its expression is an attribute of benign epithelial tumors, with increased expression triggering cSCC to shift to a benign tumor phenotype.^39^

Together our findings indicate that a direct EC-Treg cross-communication in the skin forms an important early checkpoint to contain tissue damage and carcinogenesis. This supports the growing appreciation of an evolving TME and the fact that regulation of primary tumor initiation, progression and metastasis may differ.

### Limitations of the study

Although all our data indicate that the importance of IL-33/ST2 signaling in the skin is mediated via tissue Tregs, we were not able to use the Treg-specific FoxP3^Cre^ mouse in this study, so for cell-specific knockout of ST2 we relied on the CD4^Cre^. Only very few FoxP3^−^ T cells express ST2; however, we cannot exclude that these cells may also contribute to the phenotype. Further, it is currently unclear exactly how tissue Treg contribute to *de novo* epithelial regeneration and limitation of cancer initiation. Possible pathways are discussed above but the mechanism (s) remains to be fully elucidated. Finally, although our data suggest that the IL-33/ST2 axis is involved also in human skin, and other human epithelial, cancers it remains to be explored if a similar link to tissue Tregs and early restraint of tissue damage/cancer initiation holds true in human.

## Resource Availability

### Lead contact

Further information and requests for resources and reagents should be directed to and will be fulfilled by the lead contact, Jessica Strid (j.strid@ imperial.ac.uk).

### Materials availability

Reagents and resources can be shared upon request.

### Data and code availability

- All data reported in this paper will be shared by the lead contact upon request.
- This paper does not report any new code.
- RNA sequencing data are available from the public repository on the National Center for Biotechnology Information’s Sequence Read Archive in raw format (GEO accession number: GSE241061).
- WES data are available from the Sequence Read Archive database (SRA accession number: PRJNA1006323).
- Any additional information required to reanalyze the data reported in this paper is available from the lead contact upon request.

## Star★Methods

### Key Resources Table

**Table:** 

REAGENT or RESOURCE	SOURCE	IDENTIFIER
Antibodies		
CD11b BV421/APCCy7 Conjugated (M1/70) (1/1000)	Biolegend	Cat# 101235; RRID:AB_10897942
Siglec-F BV421 conjugated (E50-2440) (1/100)	BD Biosciences	Cat# 562681; RRID:AB_2722581
Ly6C PerCPcy5.5 Conjugated (HK1.4) (1/400)	Biolegend	Cat# 128011; RRID:AB_1659242
Ly6G APCCy7 Conjugated (IA8) (1/800)	Biolegend	Cat# 127623; RRID:AB_10645331
CD4 BV605 Conjugated (GK1.5) (1/400)	Biolegend	Cat# 100451; RRID:AB_2564591
CD8 BV421 Conjugated (53−6.7) (1/200)	Biolegend	Cat# 100737; RRID:AB_10897101
CD45 APC conjugated (30-F11) (1/200)	Biolegend	Cat# 103111; RRID:AB_312976
IgE FITC/PE Conjugated (R35-72) (1/100)	BD Biosciences	Cat# 553415; RRID:AB_394848
TCRβ BV785 conjugated (H57-597) (1/200)	Biolegend	Cat# 109249; RRID:AB_2810347
TCRγβPerCPCy5.5/APC conjugated (eBioGL3) (1/500)	Thermo Fisher	Cat# 17-5711-82; RRID:AB_842756
CD117 PECy7 conjugated (2B8) (1/100)	Biolegend	Cat# 105814; RRID:AB_313223
CD11c BV711 Conjugated (HL3) (1/100)	BD Biosciences	Cat# 563130; RRID:AB_2738019
Foxp3 APC conjugated (FJK-16s) (1/50)	Thermo Fisher	Cat# 17-5773-82; RRID:AB_469457
CD103 FITC conjugated (2E7) (1/200)	Biolegend	Cat# 121419; RRID:AB_10709438
KLRG1 FITC conjugated (2F1) (1/200)	Biolegend	Cat# 138409; RRID:AB_10643998
ICOS PE/BV605 conjugated (15F9) (1/200)	Biolegend	Cat# 107705; RRID:AB_313334
CD25 PECy7 conjugated (PC61.5) (1/100)	Thermo Fisher	Cat# 25-0251-82; RRID:AB_469608
TIGIT PE conjugated (1G9) (1/100)	BD Biosciences	Cat# 565168; RRID:AB_2739089
CTLA4 PE conjugated (UC10-4F10-11) (1/400)	BD Biosciences	Cat# 561718; RRID:AB_10895585
CD69 APC conjugated (H1.2F3) (1/200)	BD Biosciences	Cat# 560689; RRID:AB_1727506
NKG2D PE conjugated (CX5) (1/100)	BD Biosciences	Cat# 558403; RRID:AB_647201
ST2 PE conjugated (RMST2-2) (1/100)	Thermo Fisher	Cat# 12-9335-82; RRID:AB_2572708
GATA3 PE conjugated (L50-823) (1/50)	BD Biosciences	Cat# 560074; RRID:AB_1645330
IRF4 PE conjugated (3E4) (1/50)	Thermo Fisher	Cat# 12-9858-82; RRID:AB_10852721
Tbet PE conjugated (4Bio) (1/100)	Biolegend	Cat# 644809; RRID:AB_2028583
RORyt PE conjugated (Q31-378) (1/50)	BD Biosciences	Cat# 562607; RRID:AB_11153137
CD45.1 FITC conjugated (A20) (1/100)	Biolegend	Cat# 110705; RRID:AB_313494
IL-10 PE conjugated (JES5-16E3) (1/100)	BD Biosciences	Cat# 561060; RRID:AB_10561692
Fc Block (2.4G2) (1/50)	BD Biosciences	Cat# 553140; RRID:AB_394655
Live/Dead Aqua Viability dye (1/100)	Thermo Fisher	L34957
HRP-conjugated donkey anti goat IgG (1/1000)	R&D	Cat# HAF109; RRID:AB_357236
Mouse anti-human IL-33 (Nessy-1) (1/500)	SCBT	sc-517600
HRP-conjugated goat anti mouse IgG	Vector labs	MP-7452
Goat anti-mouse IL-33 (1/500)	R&D	Cat# AF3626; RRID:AB_884269
Rabbit anti-β-actin (1/1000)	Cell Signaling Technologies	4967L
Alexa Fluor 555-conjugated donkey anti-goat IgG (1/1000)	Thermo Fisher	Cat# A-21434; RRID:AB_2535855
Alexa Fluor 488-conjugated donkey anti-rat (1/2000)	Thermo Fisher	Cat# A-21208; RRID:AB_2535794
Alexa Flour 647-conjugated rat anti-mouse CD49F (1/100)	Biolegend	Cat# 313609; RRID:AB_493636
Chemicals, peptides, and recombinant proteins		
TPA (phorbol 12-myristate 13-acetate)	Sigma-Aldrich	P8139
DMBA (7,12-dimethylbenzaanthracene)	Sigma-Aldrich	D3254
Liberase	Roche	5401119001
DNase1	Roche	10104159001
S1P antagonist Fingolimod	Sigma-Aldrich	FTY720
Diptheria Toxin	Sigma-Aldrich	D0564
PMA	Sigma-Aldrich	P1585
lonomycin	Sigma-Aldrich	I3909
Brefeldin A	Sigma-Aldrich	B6542
β-mercaptoethanol	Sigma Aldrich	444203
Critical commercial assays		
CD4^+^CD25^+^ Regulatory T cell Isolation Kit, mouse	Miltenyi	130-091-041
FoxP3/Transcription factor staining buffer	Thermo Fisher	11500597
Intracellular staining kit	Invitrogen	88-8824-00
RNEasy Micro kit	Qiagen	74004
RNEasy Mini kit	Qiagen	74104
iScript cDNA synthesis kit	Bio-Rad	1708890
MGIEasy Universal Library Conversion Kit	MGI Tech	1000004155
Gentra Puregene kits	Qiagen	158445
Nextera Index Kit	Illumina	FC-131-1001
Twist Mouse Whole Exome-Probe kit	Twist Bioscience	102035
xGen Hybridization & Wash kit	IDT	10010352/10010354
Deposited data		
RNAseq data	This paper	GEO accession number: GSE241061
Whole exome sequencing	This paper	SRA accession number: PRJNA1006323
Experimental models: Organisms/strains		
Mouse: IL33^cit^ (BALB/c)		Townsend et al.^41^
Mouse: IL1r11^−/−^ (BALB/c)		Townsend et al.^41^
Mouse: Tcrb^−/−^ (FVB/N)		Mombaerts et al.^42^
Mouse: Foxp3^egfp^ (C57BL/6)		Wang et al.^43^
Mouse: Foxp3^DTR^ (C57BL/6)		Kim et al.^44^
Mouse: IL1rL1^fl/fl^ (C57BL/6)		Skarnes et al.^45^
Oligonucleotides		
Icos Fp: 5’ -CAAGAAAGGAACCTTAGTGGAGGAT-3’	This paper	N/A
Icos Rp: 5’ -ACGGGTAGCCAGAGCTTCAG-3 ’	This paper	N/A
Tigit Fp: 5’ -TCCTGGTGGGATTTACAAGG-3’	This paper	N/A
Tigit Rp: 5’ -AAGCAAATGAGTCCCAGCAC-3’	This paper	N/A
CD69 Fp: 5’ -TGGTCCTCATCACGTCCTTAATAA-3’	This paper	N/A
CD69 Rp: 5’ -TCCAACTTCTCGTACAAGCCTG-3’	This paper	N/A
Areg Fp: 5’ -CAGTGCACCTTTGGAAACGA-3’	This paper	N/A
Areg Rp: 5’ -GTGACAACTGGGCATCTGGA-3’	This paper	N/A
Gzmb Fp; 5’ -ATCCTGCTCTGATTACCCATCGT-3’	This paper	N/A
Gzmb Rp: 5’ -ATGGATATGAAGCCAGTCTTTGC-3’	This paper	N/A
Krt5 Fp: 5’ -CTTGTGGAGTGGGTGGCTAT-3’	This paper	N/A
Krt5 Rp: 5’ - CCACTTGGTGTCCAGAACCT-3’	This paper	N/A
Krt14 Fp: 5’ -CAGCCCCTACTTCAAGACCA-3’	This paper	N/A
Krt14 Rp: 5’ - GGCTCTCAATCTGCATCTCC-3’	This paper	N/A
Krt1 Fp: 5’ -TTTGCCTCCTTCATCGACA-3’	This paper	N/A
Krt1 Rp: 5’ - GTTTTGGGTCCGGGTTGT-3 ’	This paper	N/A
Krt10 Fp: 5’ -GGATGAGCTGACCCTTAGCA-3 ’	This paper	N/A
Krt10 Rp: 5’ - CATTTTGAAGGTCTCTCATTTCCT-3’	This paper	N/A
Krt16 Fp: 5’ -TCTGGACAGTCCTATTCTTCTCG-3’	This paper	N/A
Krt16 Rp: 5’- CTTGCTCCTTGAGGATGGAC-3’	This paper	N/A
Krt8 Fp: 5’ -CAAGGTGGAACTAGAGTCCCG-3’	This paper	N/A
Krt8 Rp; 5’ -CTCGTACTGGGCACGAACTTC-3’	This paper	N/A
Krt18 Fp; 5’ -CAAGTCTGCCGAAA TCAGGGAC-3’	This paper	N/A
Krt18 Rp; 5’ - TCCAAGTTGATGTTCTGGTTTT-3 ’	This paper	N/A
Krt13 Fp; 5’ -CTTGCTCCCACCA TGAGCTG-3 ’	This paper	N/A
Krt13 Rp: 5’ -CCATCGACACCTCCGAAGTC-3’	This paper	N/A
Cldn Fp: 5’ -GCCATCTACGAGGGACTGTG-3’	This paper	N/A
Cldn Rp: 5’ -CACTAATGTCGCCAGACCTGAA-3’	This paper	N/A
Ocln Fp; 5’ -TTGAACTGTGGATTGGCAGC -3’	This paper	N/A
Ocln Rp: 5’ -CAAGATAAGCGAACCTTGGCG-3’	This paper	N/A
Software and algorithms		
FlowJo		https://www.flowjo.com/
ImageJ		https://imagej.net/ij/
Nanostring nSolver analysis software		https://nanostring.com/products/ncounter-analysis-system/nsolver-advanced-analysis-software/
GEPIA (Gene Expression Profiling Interactive Analysis)		http://gepia.cancer-pku.cn/
g:Profiler		https://biit.cs.ut.ee/gprofiler/page/docs
Kaplan-Meier Plotter		https://kmplot.com/analysis/
FastQC (Illumina)		https://emea.illumina.com/products/by-type/informatics-products/basespace-sequence-hub/apps/fastqc.html
Salmon package (v.1.5.2)		
Picard-collecRNAseqmetrics (v.3.0.0)		
DESeq2 (v.1.38.2)		https://bioconductor.org/packages/release/bioc/html/DESeq2.html
Nf-core pipeline Sarek (v3.1.2)		https://nf-co.re/sarek/3.4.2/
R/Bioconductor environment (v.4.2.2)		http://www.R-project.org/
GraphPad - Prism		https://www.graphpad.com/features

### Experimental Model and Study Participant Details

#### Mouse strains

Genetically altered mice were generated as previously described. *Il33*^*cit*40^ and *Il1rl1*^−/−41^ were on the BALB/c background after >10 backcrosses. *Il33*^*cit*^ were bred to homozygosity for use as IL33^−/−^. *Tcrb*^−/−42^ were on the FVB/N background after >10 backcrosses and Foxp3^egfp43^ reporter mice, Foxp3^DTR44^ and *Il1rl1*^*fl/fl*45^ mice were on the C57BL/6 background. Strain- and sex-matched control animals were purchased from Charles River or littermates controls where used. Mice were bred and maintained in individually ventilated cages under specific pathogen-free conditions; with food and water provided *ad libitum*. Age-matched, female mice were used for all experiments at ≥ 7 weeks of age and selected at random from a large pool when allocated to experiments. All studies were approved by Imperial College AWERB (Animal Welfare and Ethical Review Body) and the UK Home Office for Laboratory Animal Care regulations. Experiments involving cancer studies strictly adhered to the guidelines set out by the National Cancer Research Institute (NCRI) and Workman et al.^46^ in ‘Guidelines for the Welfare and Use of Animals in Cancer Research’. All studies using animals were conducted following the Animal Research: Reporting *In Vivo* Experiments (ARRIVE) guidelines.^47^

#### Human cSCC

The studies of human cSCC were conducted according to the Declaration of Helsinki Principles and all patients donating samples to this study provided written, informed consent in accordance with ethical approval; research ethics number: NHS REC 08.S1401.69. cSCC tumors were graded by an experienced dermatopathologist as low risk or high risk based on the Intercollegiate Guidelines Network for cutaneous SCC. In addition, peri-lesional skin (histopathologically normal looking skin >4 mm away from the tumor edge) and abdominal non-UV exposed skin were also analyzed.

## Method Details

### Cutaneous challenge and chemical carcinogenesis

Chemicals 12–0-tetradecanoylphorbol-13-acetate (TPA) and 7,12-dimethylbenz[*a*]anthracene (DMBA) were purchased from Sigma and dissolved in 100% ethanol or acetone respectively. Mice requiring topical application of chemicals to the back were shaved using hair clippers 2–3 days prior to the treatment and rested. Acute skin damage was induced by exposing the dorsal sides of the ear skin or the shaved back skin to a single or repeated dose of DMBA (ears: 200nM; back: 600nM) or TPA (ears: 2.5nM; back: 40nM).

For cutaneous carcinogenesis, age-matched female mice were used at 7 weeks of age. All mice were shaved on the back using hair clippers and allowed to rest for 2–3 days prior to DMBA initiation. Applications of chemicals and tumor monitoring were performed as previously described.^9^ In brief, for DMBA-TPA carcinogenesis, 200nM DMBA was carefully and slowly applied by pipette, in a 150mL volume, to the entire shaved skin area. Mice were rested for 1 week and 20nM TPA then applied twice weekly. For DMBA only carcinogenesis, 200nM DMBA was applied initially, mice were rested for 1 week and 100nM DMBA then applied weekly. Hair regrowth during the experiments was gently removed by clipping with trimmers. Mice were monitored daily, and cutaneous tumors were counted and measured with a caliper once weekly. Back skin and tumors were evaluated by visual inspection by an observer blinded to the experimental groups.

### Tissue processing

Skin and tumor tissues were cut into small 1 mm^2^ pieces using a scalpel blade and incubated for 90mins in digestion buffer containing 25ug/ml liberase (Roche), 250ug/ml DNAseI (Roche) and 1x DNAse buffer (1.21g Tris base, 0.5g MgCl_2_ and 0.073g CaCl_2_) at 37°. Following digestion, tissue was transferred into C-tubes (Miltenyi Biotech) containing RPMI-1640 media (Thermo Fisher) supplemented with 10% heat-inactivated fetal calf serum, 1% penicillin-streptomycin-glutamine (Thermo Fisher) and physically disrupted using a Miltenyi cell dissociator program 01-B three times. All cell suspensions were filtered through a 70mm filter and cells were counted using a CASY cell counter (Roche).

### Flow cytometry and cell sorting

Prior to all staining protocols, cell suspensions were blocked for non-specific antibody binding using blocking buffer containing flow cytometry (FC) buffer (1x PBS, 10% FCS, 0.09% Sodium Azide and 2mM EDTA), 2% normal rat serum (Sigma Aldrich) and 2% Mouse BD Fc Block (2.4G2) and incubated for 30 min at 4°C.

For cell surface staining, cell suspensions were stained with LiveDead Aqua (Thermo Fisher Scientific) and cell surface antibodies, diluted to the appropriate concentrations in FC buffer, and incubated at 4°C for 30mins and subsequently washed with FC buffer. For transcription factor antibody staining, the FoxP3/Transcription factor staining buffer set (Thermo Fisher Scientific) was used as per instructions. In brief, following cell surface antibody staining, cell suspensions were fixed with 1x Foxp3 fixation/diluent solution (4xFoxP3 fixation reagent diluted with FoxP3 diluent reagent) for 1 h at 4°C. Cell suspensions were washed twice in 1x permeabilization buffer and were resuspended in blocking buffer (1xpermeabilization buffer with 2% NRS) for 15 mins at 4°C. Without washing, the transcription factor antibodies and appropriate isotype controls were directly added at the appropriate dilution for at least 30mins and subsequently washed twice with 1xpermeabilization buffer and once with FC buffer. For intracellular cytokine staining, cells were incubated with PMA (Sigma Aldrich) and ionomycin (Sigma Aldrich) (50 ng/ml and 1ug/ml respectively) for 2hrs before addition of 10ug/ml brefeldin A (Sigma Aldrich) for a further 4hrs. Cells were washed, surface stained and then fixed using the Intacellular Fixation and Permabilization buffer set (Invitrogen) prior to intracellular antibody stain. All antibody-stained cell suspensions were analyzed on a BD LSR Fortessa X-20 flow cytometer with BD FACSDIVA software (BD). The data was further analyzed using FlowJo software (BD).

For cell sorting, antibody-stained single cell suspensions were fluorescence activated cell sorted (FACS) using a FACS Aria III High Speed Cell Sorter machine. The cells were sorted straight into RLT buffer (Qiagen) supplemented with 1% b-mercaptoethanol (Sigma Aldrich). CD4^+^Tregs were sorted from tissues using the Foxp3^egfp^ reporter strain. Where Foxp3^egfp^ reporter mice were not suitable, CD4^+^Tregs in the skin were sorted as TCRb^+^CD4^+^CD103^+^CD25^+^ and CD4^+^ non-Tregs as TCRb^+^CD4^+^CD103^+^CD25^−^.

Tissue cells subsets were gated on live singlets for all and then as: mast cells: CD45^+^CD117^+^IgE^+^; basophils: CD45^mid^CD117^−^IgE^+^; eosinophils: CD45^+^CD11b^+^Siglec-F^+^; neutrophils: CD45^+^Ly6G^+^Ly6C^−^CD11b^+^; macrophages: CD45^+^CD11b^+^Ly6G^−^Ly6C^−^F4/80^+^; mopnocytes: CD45^+^CD11b^+^Ly6G^−^Ly6C^+^F4/80^−^; γδT cells: CD45^+^CD3^+^Tcrd^+^; αβT cells: CD45^+^CD3^+^Tcrb^+^CD4^+^ or CD8^+^; Tregs: CD45^+^CD3^+^Tcrb^+^CD4^+^Foxp3^+^; Non-Tregs: CD45^+^CD3^+^Tcrb^+^CD4^+^Foxp3^−^; NK cells: CD45^+^Tcrb^−^Tcrd^−^NKG2D^+^; CD11c^+^DCs: CD45^+^Ly6G^−^Ly6C^−^Siglec-F^−^Tcrb^−^Tcrd^−^NKG2D^−^F4/80^−^MHCII^+^CD11c^+^; ILC2s: Tcrb^−^Tcrd^−^NKG2D^−^F4/80^−^CD11b^−^MHCII^−^CD11c^−^ IgE^−^CD127^+^CD103^+^.

Details of all antibodies used can be found in the “key resources table”.

### Adoptive transfer experiments

Adoptive cell transfers were carried out in one of two ways, according to the experimental question outlined in the text: 1) total lymphocytes were isolated from the local skin draining lymph nodes of CD45.1 donor mice and 4×10^6^ cells were transferred by i.v. injection into CD45.2 recipient mice or 2) CD3^+^CD4^+^CD25^+^ Tregs were purified from the spleen of WT donors using Miltenyi Biotech isolation kits (Cat. no: 130-091-041). Treg purity was checked by Foxp3/CD25 FACS staining and 0.5-1×10^6^ Treg were transferred i.v. into *Tcrb*^−/−^ recipients.

### FTY720 and diphtheria toxin administration

To sequester lymphocytes in the LN by blocking egress, the S1P antagonist Fingolimod (FTY720; Sigma Aldrich) was injected i.p at 3 mg/kg 24h prior to topical exposure to DMBA and then daily till the end of the experiment (7 days).

To deplete Tregs in FOXP3^DTR^ transgenic mice, 0.25mg diphtheria toxin was injected i.p. 3x a week for short (7–10 days experiments) and once weekly for longer experiments (max 6 weeks).

### Western blotting

Ear or back skin was floated dermal side-down in TrypLE Express (Thermos Fisher Scientific) for 2hrs, 37°C, 5% v/v CO_2_. The epidermis was removed and further digested for a single cell suspension in TrypLE Express solution with 200 μg/ml DNAse I (Roche) and 1xDNAse buffer on a rotator for 30 mins at 37°C, 5% v/v CO_2_. Epithelial cell suspensions were lysed using RIPA Buffer (Sigma Aldrich) supplemented with 10μL 50mM phenylmethylsulphonyl fluoride (PMSF; Sigma Aldrich), 10μL phosphatase cocktail (Sigma Aldrich), 20μL protease cocktail (Sigma Aldrich), 50μL 1M sodium fluoride (Sigma Aldrich), 1μL 1M sodium orthovanadate (Na_3_VO_4_; Sigma Aldrich) for 1hr on ice and subsequently sonicated three times for 10s. Protein supernatants were collected after centrifugation and protein concentrations were determined by Bradford assay (1μL of protein lysate was added to 200μL of Bradford reagent and absorbance was read at 595nm).

Protein lysates (25μg) were boiled (95°C) for 5mins in NuPage LDS loading buffer supplemented with 5% β-mercaptoethanol (Sigma Aldrich) and ran on a 12% SDS-PAGE gel. The proteins were electroblotted for 1 h at 300mA onto a 0.45μm polyvinylidene difluoride (PVDF) membrane (Millipore). Prior to staining non-specific staining was blocked with 5% milk (Marvel) in TBS-0.1% Tween

20 for 1 h at RT. Membrane were probed with Goat anti-IL-33 (AF3626; R&D) or Rabbit anti-b-actin (4967L; Cell signaling technologies) primary antibodies overnight at 4°C in blocking buffer. Secondary antibodies used were HRP conjugated donkey anti Goat IgG (R&D) or HRP conjugated swine anti rabbit IgG in blocking buffer for 1 h at RT. Membranes were washed in between stains with 0.1% Tween 20-TBS. Proteins were visualized using Pierce ECL Western Blotting Substrate and Amersham Hyperfilm ECL (GE Healthcare Life Sciences). Antibodies were stripped from the membrane using Restore Western Blot Stripping Buffer (Thermo Fisher) as per manufacturer’s instructions.

### Immunofluorescence staining of mouse skin and tumor samples

For ear skin, ears were removed, and a defined central section cut. Tumors were removed from the back skin along with a small piece of adjacent skin. All tissues were snap-frozen in OCT on dry ice. 5-8μm sections were cut using a Leica JUNG CM1800 cryostat and stored at -80°C. For staining, slides were brought back up to RT before fixation with 4% paraformaldehyde for 15mins. Samples were washed in PBS before permed with PBS+0.2% Triton x100 for 15 mins at RT. Samples were then blocked in 5% donkey serum for 1 h at room temperature before staining with primary antibody overnight at 4°C. Primary antibodies: goat anti-mouse IL-33 antibody (AF3626; R&D systems), rat anti-mouse CD45 (30-F11; Biolegend) or rat-anti FoxP3 (FJK-16s; Thermo Fisher). Following staining, samples were washed with PBS+0.2% Triton x100 and incubated with the appropriate secondary antibody. Secondary antibodies: Alexa Fluor 555–conjugated donkey anti-goat IgG (A21434; ThermoFisher) and Alexa Fluor 488-conjugated donkey anti-rat (A21208; Thermo Fisher). After extensive washing, samples were then either further incubated with Alexa Flour 647-conjugated rat anti-mouse CD49F (GoH3, BioLegend) or directly mounted in VectaShield containing DAPI (Vectashield). Tissue samples were visualised with a Leica SP5 confocal laser-scanning microscope (Leica).

### Intravital imaging

Intravital microscopy was performed using a LeicaSP5 confocal microscope on the ear dermis, as previously described.^48^ Briefly, mice were anesthetized by intraperitoneal injection of ketamine/medetomidine (125/1 mg/kg). Mice were kept at 37°C and received oxygen (0.5 L/min). Ears of FoxP3^egfp^ reporter mice were taped to the center of the coverslip and 80μL of TRITC conjugated 70kDa dextran (70μM) was injected intravenously. Light was generated from 488-nm and 543-nm laser lines, and emitted light signals were detected to generate three-color 8-bit images, using a 20x/0.75 Plan Apochromat objective. Stacks of 3 squared x-y sections with 10μm z spacing were acquired. Videos were generated using ImageJ software (v1.42, NIH, USA), by summing signal intensity from each stack. Images were acquired for at least 45mins post injection. Data presented are calculated cellular behaviors using Imaris (Oxford Instruments, USA), as previously described.^49^

### Immunohistochemistry of human cSCC

cSCC tissue samples were formalin-fixed and paraffin-embedded and 5μm tissue sections were cut and placed onto 3-aminopropyltriethoxysilane-coated microscope slides. Sections were deparaffinised in xylene and rehydrated through graded alcohols. Antigen retrieval was performed with pH 6.0 sodium citrate treatment at 95 °C for 15 min and slides were permeabilised for 15mins with PBS+0.2% Triton x100. Non-specific binding was blocked with 10% normal goat serum and 1% BSA in PBS+0.1% Tween 20. Slides were stained with mouse anti-human IL-33 antibody overnight at 4C (Nessy-1; SCBT) followed by staining with HRP-conjugated goat anti mouse (MP-7452; Vector labs). Slides were washed in between stains with PBS+0.1% Tween 20. Staining was visualised with DAB substrate and brown chromogen precipitation. Slides were counter stained in Harris’ haematoxylin acid solution, dehydrated with increasing ethanol gradient and Xylene and mounted with Pertex mounting. Slides were visualised using a Leica Aperio CS2 slide scanner and Aperio ImageScope software (Leica).

### Primary neonatal keratinocyte cultures

Total body wall skin from neonatal mice (<5 days old) was incubated overnight at 4°C in 5 U/ml Dispase (BD) supplemented with 1x antibiotic and antimycotic solution (Sigma). The epidermis was isolated and further digested in TrypLE Express supplemented with 200 μg/mL DNAse I and DNAse buffer. Cell suspensions were filtered and resuspended in defined KC serum-free medium with supplements (Life) and 1x antibiotic–antimycotic solution. Keratinocytes were seeded at an appropriate cell density onto tissue culture vessels coated with rat tail-derived collagen I (Sigma). Culture vessels were washed with PBS 24 h following seeding to remove unattached cells and provided with fresh medium ± addition of conditioned media from WT or *Il1rl1*^−/−^ Treg stimulated 24h with 20 ng/ml IL-33.

### qRT-PCR

RNA was extracted from FACs sorted cell suspensions in RLT or from epidermis/tumor tissue preserved in RNA-later, using RNEasy Mini kit or RNEasy Micro kit (Qiagen) as per manufacturer’s instructions. RNA was dissolved in nuclease-free water, and yield and purity were determined. Complementary DNA (cDNA) was synthesised from RNA using the iScript cDNA synthesis kit (Bio-Rad) as per the manufacturer’s instructions. cDNA was diluted in nuclease-free double-deionized water for qRT–PCR. All primers were single-stranded DNA oligonucleotides (Sigma) that were intron-spanning, as verified by NCBI Primer-Blast tool. Real-time PCR products were detected with SYBR Green (Life) measured continuously with a ViiA 7 Real-Time PCR system (Applied Biosystems, CA, USA). Ct values for genes of interest were normalised against Ct values of the housekeeping gene Cyclophilin (Cyc) using the 2^-ΔCt^ method.

All primers used are listed in ‘‘key resources table‘‘.

### Nanostring nCounter

For human cSCC tumor gene expression, total RNA was extracted from collected samples using Qiagen RNeasy extraction kit and directly hybridized using the NanoString PanCancer and PanCancer Immune expression panel and analyzed on a nCounter (NanoString).

For mouse Treg gene expression, CD4^+^Tregs were sorted from the skin draining lymph nodes and skin of Foxp3^egfp^ reporter mice. Cells were sorted directly into RLT buffer (Qiagen) supplemented with 1% b-mercaptoethanol (Sigma Aldrich) and RNA extracted using the RNEasy Micro kit (Qiagen) as per manufacturer’s instructions. A minimum of 50,000 cells were isolated from skin tissue per sample and 1×10^6^ cells from skin draining lymph nodes. Transcriptional analysis was carried out on RNA samples using the Nanostring nCounter platform with the Mouse Immuno Profiling CodeSet (NS_Immunology_MM_C2269). Statistical analysis was performed using nSolver Analysis Software 3.0 with nCounter Advanced Analysis (version 3.0.22). Normalization and differential expression were performed using nSolver advanced analysis.

### RNA sequencing and analysis

Total RNA was extracted from whole skin biopsies using RNEasy kits (Qiagen). RNA quality control, library prep and RNA sequencing were carried out by the Imperial BRC Genomics Facility. The next generation Illumina platform HiSeq4000 was used for all samples.

For RNAseq analysis, the reads quality was checked using FastQC (v0.12.1) and summarized with MultiQC (v1.11). Reads were aligned to the FVB/NJ mice transcriptome (Mus_musculus_Fvb_Nj.FVB__NJ_v1.cdna.all.fa) using Salmon package (v.1.5.2). An average of 81.5% mapping percentage was achieved and the average number of properly aligned reads was 22 million reads. Mapping quality, read distribution, gene body coverage, GC content, and rRNA contamination, were checked using picard-collecRNA-seqmetrics (v. 3.0.0) software. Transcript level read counts were computed using Salmon package (v.1.5.2). Gene-level counts were generated from Salmon output using tximport (v1.26.1). Genes with fewer than ten aligned reads across all samples were filtered out as lowly expressed genes, keeping 15,450 expressed genes out of 26,807 total genes. Differential gene expression analysis between groups was performed using DESeq2 (v.1.38.2) and significantly differentially expressed genes were defined as fold-change at more or less than 2 and below 1% Benjamini–Hochberg (BH) adjusted *p*-value. Pathway analysis was done using g:Profiler (https://biit.cs.ut.ee/gprofiler/gost) against selected databases for terms (GO:BP, KEGG, Reactome, WikiPathway), filtering for term size 25 to 500 genes to select for the most biologically meaningful terms. Significantly enriched pathways were defined to have Bonferroni-adjusted *p*-values less than 0.01. All raw RNAseq data processing steps were performed in Cx1 high-performance cluster computing environment, Imperial College London. Further analyses were conducted in R/Bioconductor environment v.4.2.2 (http://www.R-project.org/).

### Whole exome sequencing

DNA was extracted from whole 5mm skin biopsies using Gentra Puregene kits according to manufacturer’s instruction (Qiagen). For DNA library preparation, 50ng of each sample was tagmented using in-house produced Tn5 enzyme^50^ in 1x tagmentation buffer (10 mM Tris-HCl pH 7.5, 5 mM MgCl2 and 5% dimethylformamide). Tagmentation reactions were quenched with the addition of 0.2% SDS (Thermo Fisher). Libraries were indexed using the Nextera Index Kit (dual-index; Illumina). After indexing, libraries were cleaned up using Ampure XP beads (1:1 ratio), and equimolarly pooled. For exome enrichment, 1ug of pooled libraries were mixed with Mouse Cot-1 DNA (Thermo Fisher) and xGen Universal Blockers NXT (IDT) to block unspecific probe binding. The enrichment was performed using the Twist Mouse Whole Exome-Probe Kit (Twist Bioscience) and the xGen Hybridization and Wash Kit (IDT) according to manufacturer’s instructions. For DNA library sequencing, 50ng of final exome-enriched library was converted to circular single-stranded DNA using the MGIEasy Universal Library Conversion Kit (MGI Tech) according to the manufacturer’s protocol. Next, 60fmol of circular ssDNA library pool was used for DNA nanoball (DNB) making using a custom rolling-circle amplification primer (5^′^-TCGC CGTATCATTCAAGCAGAAGACG-3^′^). Resulting DNA nanoballs were loaded onto an FCL flow cell and sequencing in PE100 mode on the G400RS sequencing platform (MGI Tech).

For data processing and analysis, Raw fastq files were demultiplexed using deML^51^ and Nextera Tn5 adapter sequences trimmed using cutadapt.^52^ Variant calling was performed using the nf-core^53^ pipeline Sarek version 3.1.2,^54^ using the GATK tool haplotypecaller^55^ to jointly call variants against genome version GRCm38. Variants from the Mouse Genomes Project^56^ from strains 129P2, 129S1, 129S5, C57BL/6NJ, and FVB/NJ were removed from the dataset as known background.^57^ Additionally, any variant present in any of the naive untreated samples were removed to focus on *de novo* variants following carcinogen exposure. Remaining variants were further filtered to keep only variants with a PASS filter from haplotypecaller and a position read depth of at least 5.

### Quantification and Statistical Analysis

#### Statistical evaluation

The statistical significance of difference between experimental groups was determined using two-tailed Student’s t-test for unpaired data, one-way ANOVA multiple comparison, Log rank Mantel-Cox test or linear regression, where appropriate, with results deemed significant at *p* < 0.05. Stars of significance correlate to: *p < 0.05; **p < 0.01; ***p < 0.001 and ****p < 0.0001. Statistics was performed with GraphPad Prism 6.00 for Mac (GraphPad; La Jolla, CA, USA).

#### Key to tumor abbreviations

ACC – Adrenocortical carcinoma; BLCA - Bladder Urothelial Carcinoma; BRCA - Breast invasive carcinoma; CESC - Cervical squamous cell carcinoma and endocervical adenocarcinoma; CHOL - Cholangio carcinoma; COAD - Colon adenocarcinoma; DLBC - Lymphoid Neoplasm Diffuse Large B-cell Lymphoma; ESCA - Esophageal carcinoma; GBM - Glioblastoma multiforme; HNSC - Head and Neck squamous cell carcinoma; KICH - Kidney Chromophobe; KIRC - Kidney renal clear cell carcinoma; KIRP - Kidney renal papillary cell carcinoma; LAML - Acute Myeloid Leukemia; LGG - Brain Lower Grade Glioma; LIHC - Liver hepatocellular carcinoma; LUAD - Lung adenocarcinoma; LUSC - Lung squamous cell carcinoma; MESO – Mesothelioma; OV - Ovarian serous cystadenocarcinoma; PAAD - Pancreatic adenocarcinoma; PCPG - Pheochromocytoma and Paraganglioma; PRAD - Prostate adenocarcinoma; READ - Rectum adenocarcinoma; SARC – Sarcoma; SKCM - Skin Cutaneous Melanoma; STAD - Stomach adenocarcinoma; TGCT - Testicular Germ Cell Tumors; THCA - Thyroid carcinoma; THYM – Thymoma; UCEC - Uterine Corpus Endometrial Carcinoma; UCS - Uterine Carcinosarcoma; UVM - Uveal Melanoma.

### Additional Resources

The data supporting the findings of this study are available from the corresponding author upon request. RNA sequencing data are available from the public repository on the National Center for Biotechnology Information’s Sequence Read Archive in raw format (GEO accession number: GSE241061). WES data are available from the Sequence Read Archive database (SRA accession number: PRJNA1006323).

## Supplementary Material

Supplemental information can be found online at https://doi.org/10.1016/j.celrep.2025.115837.

Supplementary file

## Highlights

- IL-33 expression distinguishes healthy from malignant skin
- The IL-33/ST2 axis protects against carcinogenesis
- Skin Tregs reduce mutational load and restrain cancer initiation

## In brief

Ward and Crawford et al. demonstrate that IL-33 is lost from malignant skin epithelia and that its receptor, ST2, is predominantly expressed on skin Tregs. IL-33/ST2 signals tissue damage to skin Tregs, which promote *de novo* epithelial development and thus decrease the mutational load and restrain carcinogenesis upon exposure to environmental carcinogens.
