# Supplementary material for: The IL-33/ST2 axis and tissue Treg maintain epithelial homeostasis and restrain cancer development in the skin
Source: Cell Rep. Author manuscript; Available in PMC 2026 Jul 14. (PMC7619240; doi:10.1016/j.celrep.2025.115837)
Supplement: Supplementary file [file EMS214221-supplement-Supplementary_file.pdf]

Supplementary figure 1

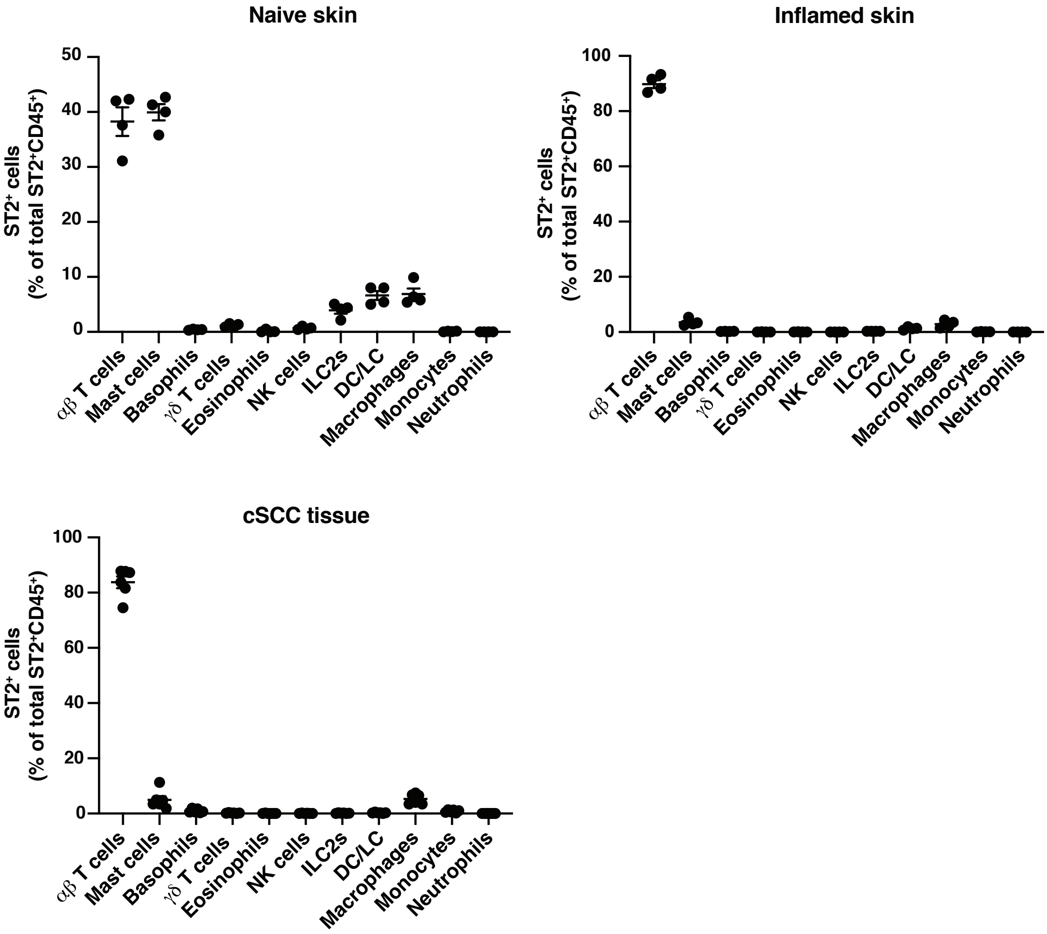

Supplementary figure 1 | ST2 expression on skin tissue immune cells

Detailed flow cytometric analysis of ST2 expression on skin leukocyte subpopulations from UT skin (n=4), inflamed skin (induced by 2x topical exposure to DMBA three days apart and analysed 1 week later) (n=4) or cSCC tissue (induced by DMBA-TPA carcinogenesis and analysed at 18 weeks) (n=6) from WT mice. Data shows ST2 expressing cell subsets as a proportion of total live $CD45^{+}ST2^{+}$cells and are represented as mean ± SEM. Gating strategy for all immune subpopulations is delineated in the 'methods' section.

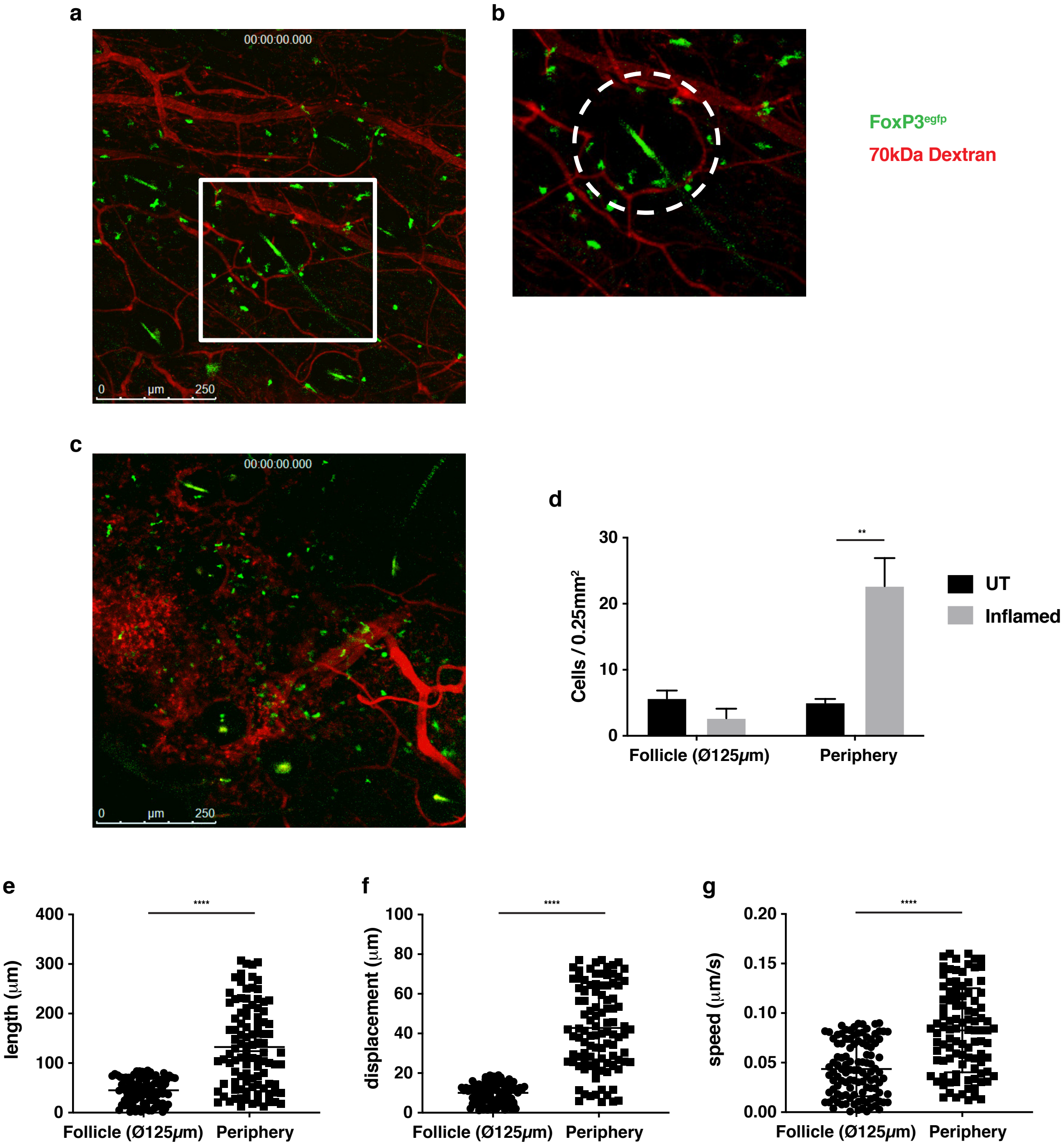

Supplementary figure 2 | FoxP3^{+} cells are abundant in healthy skin tissue and display a slow scanning movement around hair follicles, whilst FoxP3^{+} cells in inflamed skin accumulate outside follicles and have enhanced motility

Intravital time-lapse imaging of healthy UT ear skin (a,b) and inflamed ear skin (topical TPA) (c) in FoxP3^{egfp} reporter mice was performed using an inverted Leica TC5 confocal microscope. Maximum intensity projections of time-lapse imaging were generated and analysed using the Bitplane Imaris software package. FoxP3^{+} Treg are shown in green and the vasculature in red. To visualise the vasculature, 5mg of TRITC-conjugated 70kDa Dextran was injected i.v. at the start of imaging. (d) A 0.25mm^2 region of interest was drawn and FoxP3^{+} cells were counted within or outside (denoted periphery) a 125µm diameter around the hair follicle (n=3 per group). Region of interest exemplified in (a) and hair follicle diameter in (b). (e-g) FoxP3^{+} cells within or outside (periphery) the hair follicle diameter were tracked with length (e) representing the total distance moved per cell, displacement (f) referring to the straight line distance between the start and end of a tracked cell path and speed (g) showing the mm moved per second. Each dot represents one cell (n=100) and data are presented as mean ± SEM. Statistics by two-tailed unpaired Student's t-test (d-g); ** p<0.01 and **** p<0.0001.

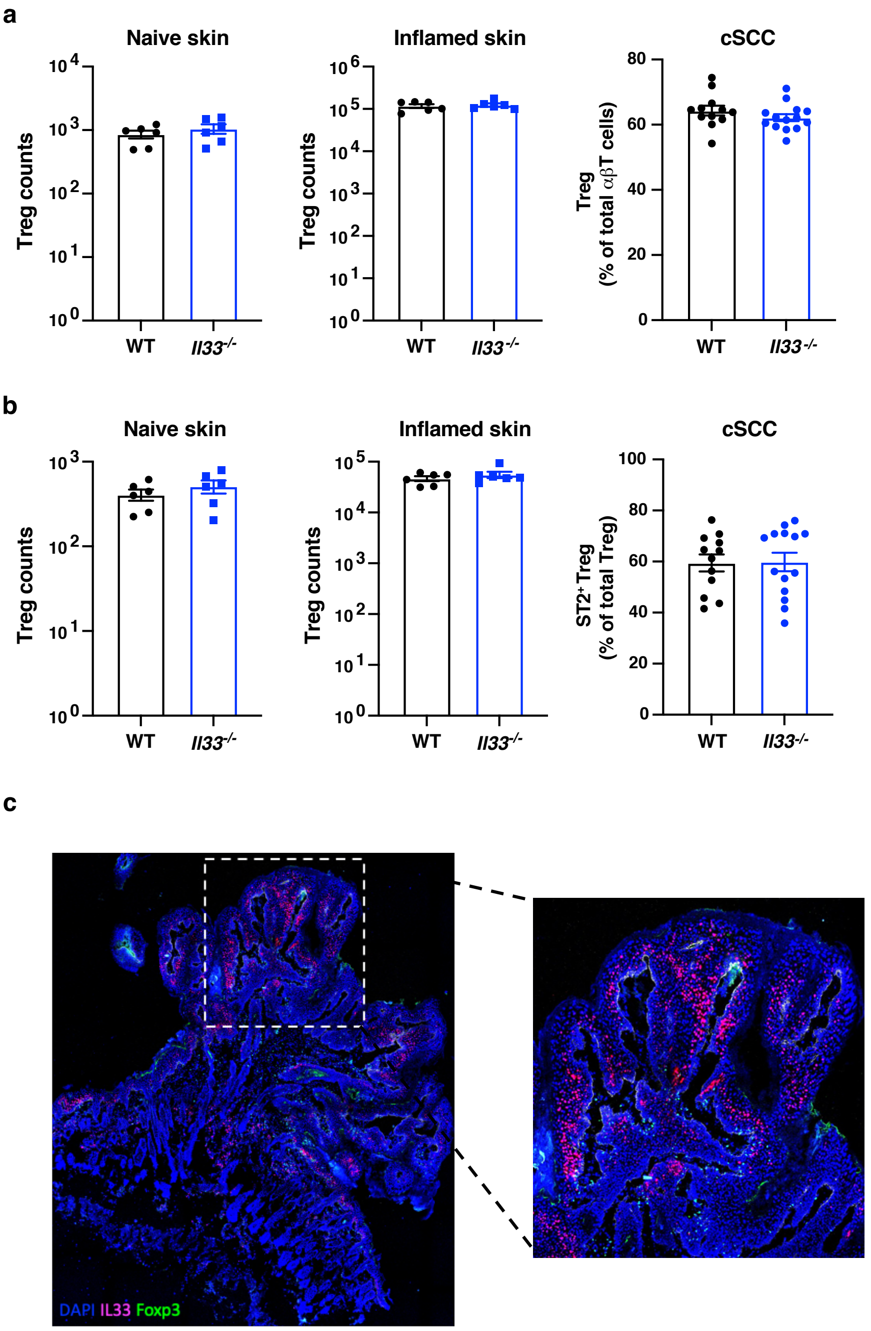

Supplementary figure 3 | Lack of the IL-33/ST2 axis does not affect Treg accumulation or localisation in the skin

(a,b) Flow cytometric analysis of (a) total Treg abundance and (b) $ST2^+$ Treg abundance in UT skin (n=6), inflamed skin (induced by topical TPA exposure) (n=6) and cSCC tissue (induced by DMBA-TPA carcinogenesis) (n=12 for WT; n=14 for *Il33*$^{-/-}$). Data are shown as Treg cell counts in UT and inflamed skin and as percentage of Treg relative to (a) total αβ T cells or (b) total Treg in cSCC samples. Treg were gated as (a) live $CD45^+Tcrb^+CD4^+FoxP3^+$ and (b) live $CD45^+Tcrb^+CD4^+FoxP3^+ST2^+$ and the data are represented as mean ± SEM. (c) Representative image of cSCC from ST2-deficient (*Il1rl1*$^{-/-}$) mouse showing IL-33 (red), FoxP3 (green) and nuclei (blue). Image taken by confocal microscopy using x20 tile scan with indicated area magnified in additional image.

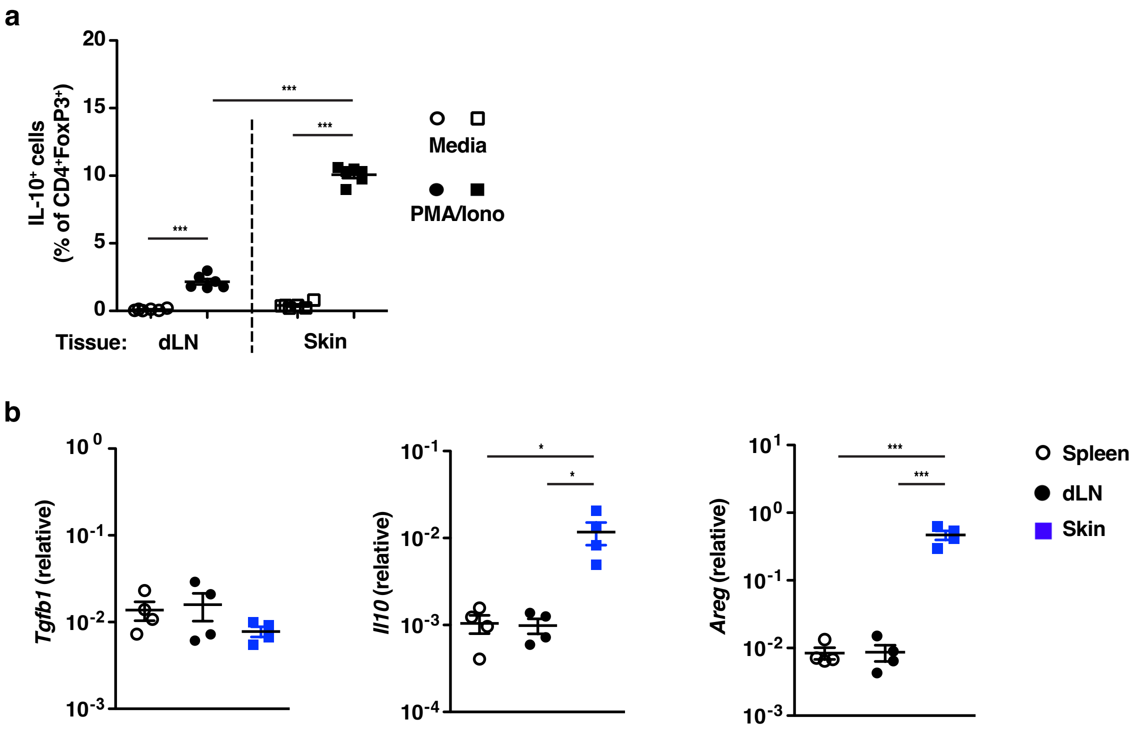

Supplementary figure 4 | Skin Treg express high levels of IL-10 and amphiregulin

The dorsal ear skin of WT mice was topically exposed to the carcinogen DMBA twice, 3 days apart and the skin, skin dLNs and spleen analysed 7 days later. (a) Lymphocytes isolated from skin or dLN were stimulated *ex vivo* with PMA/ionomycin or media alone for 6h and intracellular IL-10 analysed by flow cytometry. Graph depicts IL-10^{+} Treg as proportion of all $CD4^{+}FoxP3^{+}$ T cells (n=6). (b) $CD4^{+}egfp^{+}$ Treg were sorted by FACS from skin, dLN and spleen of $FoxP3^{egfp}$ reporter mice, total RNA extracted and the expression of *Tgfb1*, *Il10* and *Areg* was assessed by qRT-PCR relative to the housekeeping gene cyclophilin (n=4). Statistics by unpaired students t-test; * $p<0.05$ and *** $p<0.001$.

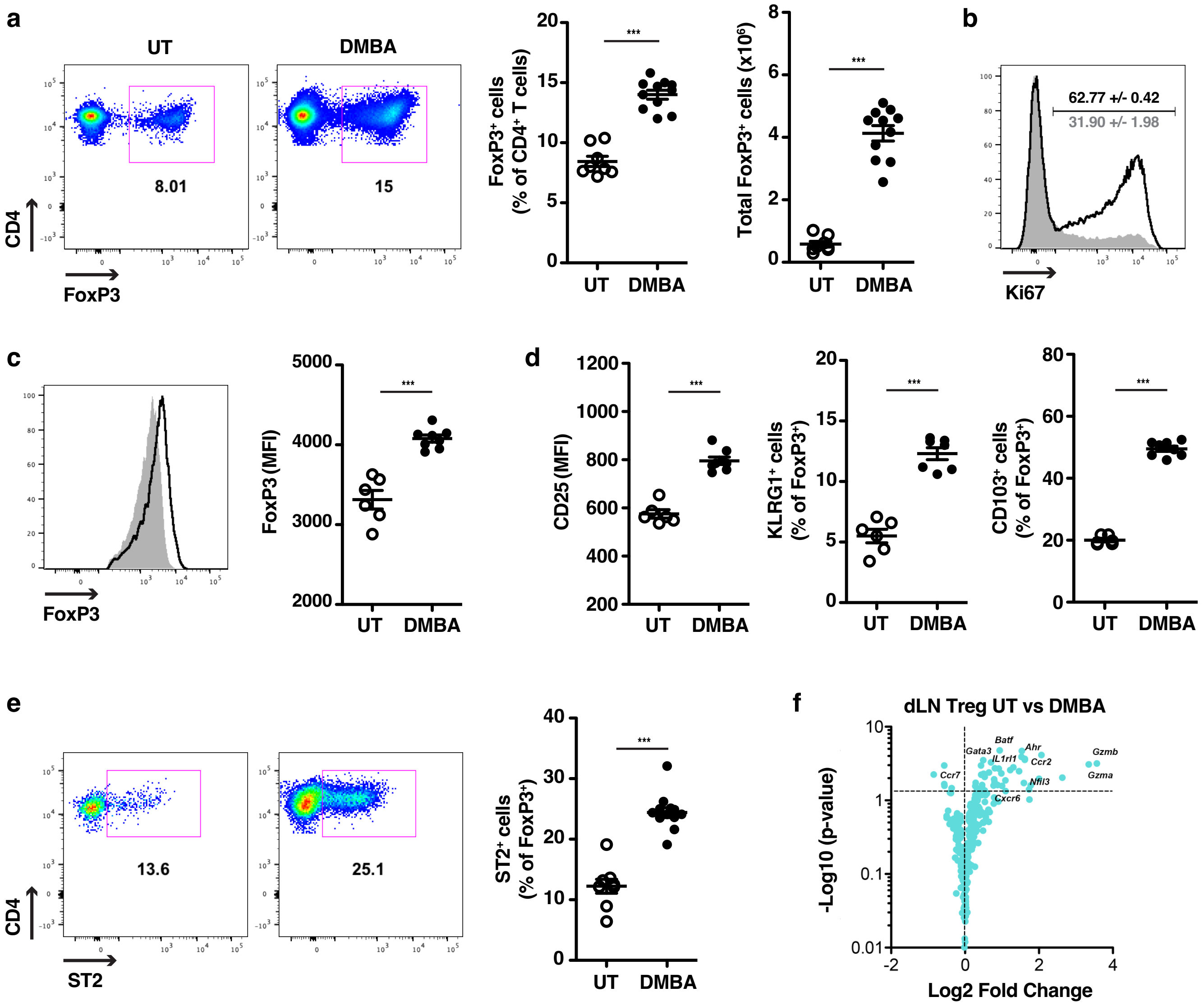

Supplementary figure 5 | Topical carcinogen primes Treg in the skin dLNs for expansion and ST2 expression

The dorsal ear skin of WT mice was topically exposed to the carcinogen DMBA twice, 3 days apart and the skin dLNs analysed 7 days later by flow cytometry (a-e) and NanoString^{TM} (f). (a) Representative dotplots showing CD4$^+$FoxP3$^+$ T cells in the dLN of naïve UT (n=6) and DMBA treated (n=11) mice with graphs depicting the relative proportion of CD4$^+$ T cells expressing FoxP3 and the total number of FoxP3$^+$ T cells in the dLN. (b) Typical histogram showing Ki67 staining of CD4$^+$FoxP3$^+$ T cells from the dLNs of UT (grey fill) and DMBA treated (black line) mice. Mean and standard deviation shown on plot. (c) Representative histogram showing FoxP3 expression level (MFI) of CD4$^+$ T cells in dLN of UT (grey fill) and DMBA treated (black line) mice and graph demonstrating FoxP3 MFI (UT n=6, DMBA n=8). (d) Analysis of activation and tissue residency markers CD25, KLRG1 and CD103 on FoxP3$^+$ Treg in dLN of UT (n=6) or DMBA treated (n=8) mice shown as MFI (CD25) and % of FoxP3$^+$ cells (KLRG1 and CD103). (e) Representative dotplots illustrating expression of ST2 on Treg in dLNs from UT (n=9) or DMBA treated (n=16) mice with graph quantifying proportion of ST2$^+$ cells as % of total FoxP3$^+$ cells. (f) Gene expression analysis using the NanoString nCounter panel NS_Immunology_Mm_C2269. Volcano plot illustrates differentially expressed genes in dLNs Tregs from UT or DMBA treated (n=3) mice. Horizontal dotted line represents threshold for statistical significance, $p<0.05$, with specific genes of interest highlighted. Each symbol (a, c-e) represents an individual mouse with horizontal bars indicating mean ± SEM. Statistics by two-tailed unpaired Student's t-test (a, c-e) *** $p<0.001$. Data are representative of four (a-c,e) and two (b) independent experiments with similar results.

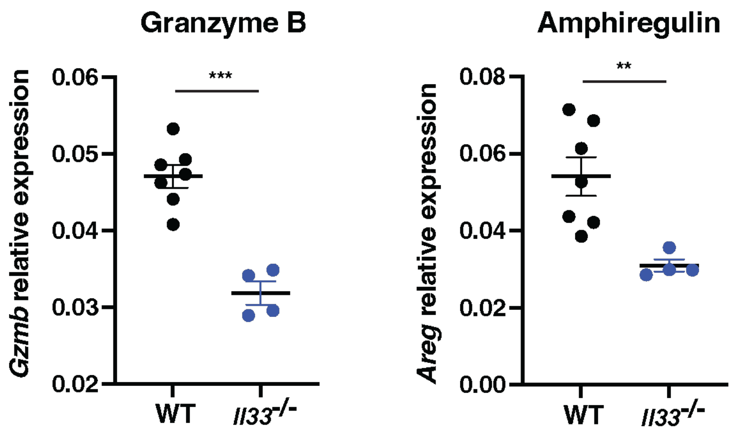

Supplementary figure 6 | Tumour Treg from *Il33*$^{-/-}$ mice are less activated

TCRβ^{+}CD4^{+}CD25^{hi}CD103^{+} Treg were sorted by FACS from cSCCs on WT (n=7) and *Il33*$^{-/-}$ (n=4) mice. Tumours were induced by DMBA-TPA inflammation-driven carcinogenesis and collected at week 20. Total RNA was extracted from Treg and gene expression of indicated genes were assessed by qRT-PCR and expressed relative to the housekeeping gene cyclophilin (*Cyc*) using $2^{-\Delta Ct}$. Data is representative of two independent experiments with similar results. Statistics by unpaired t test; ** $p<0.01$ and ***$p<0.001$.

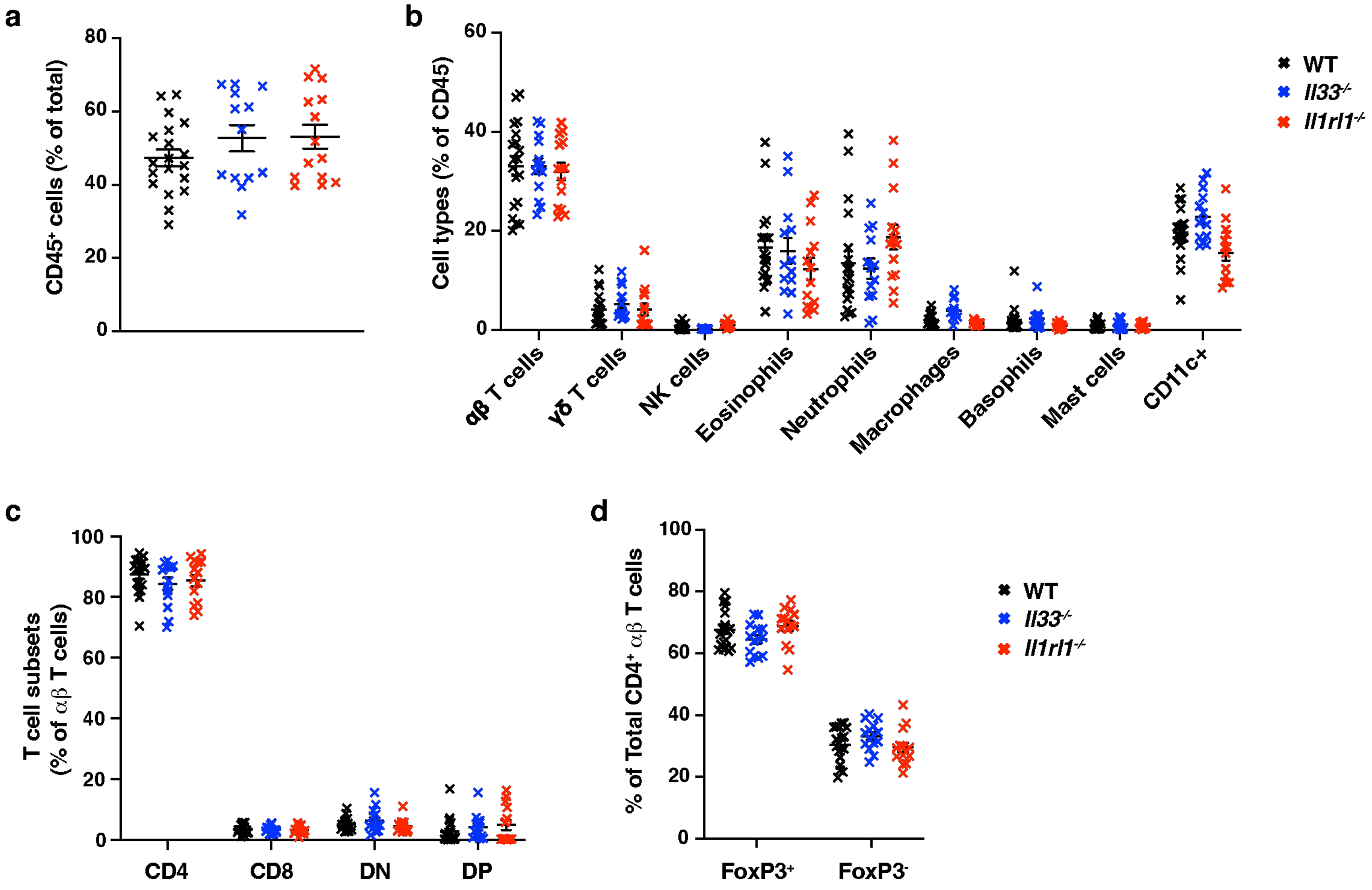

Supplementary figure 7 | The abundance or relative distribution of the tumour leukocyte infiltrate does not differ in the absence of IL-33 or ST2

cSCCs was induced by DMBA-TPA carcinogenesis on WT (n=19), *Il33*$^{-/-}$ (n=13) and *Il1rl1*$^{-/-}$ (n=14) mice. Tumours were collected at 20 weeks and the leukocyte infiltrate analysed by flow cytometry. (a) Proportion of total CD45^{+} leukocytes in the tumour tissue as % of live cells in the tumour. (b) Major lymphoid and myeloid subpopulations in the tumour tissue presented as the proportion of total CD45^{+} leukocytes. (c) Tumour αβ T cells further divided into subsets and presented as proportion of total αβ T cells and (d) the CD4^{+} αβ T cells divided into FoxP3^{+} Treg and FoxP3^{-} effector cells as a proportion of total CD4^{+} αβ T cells.

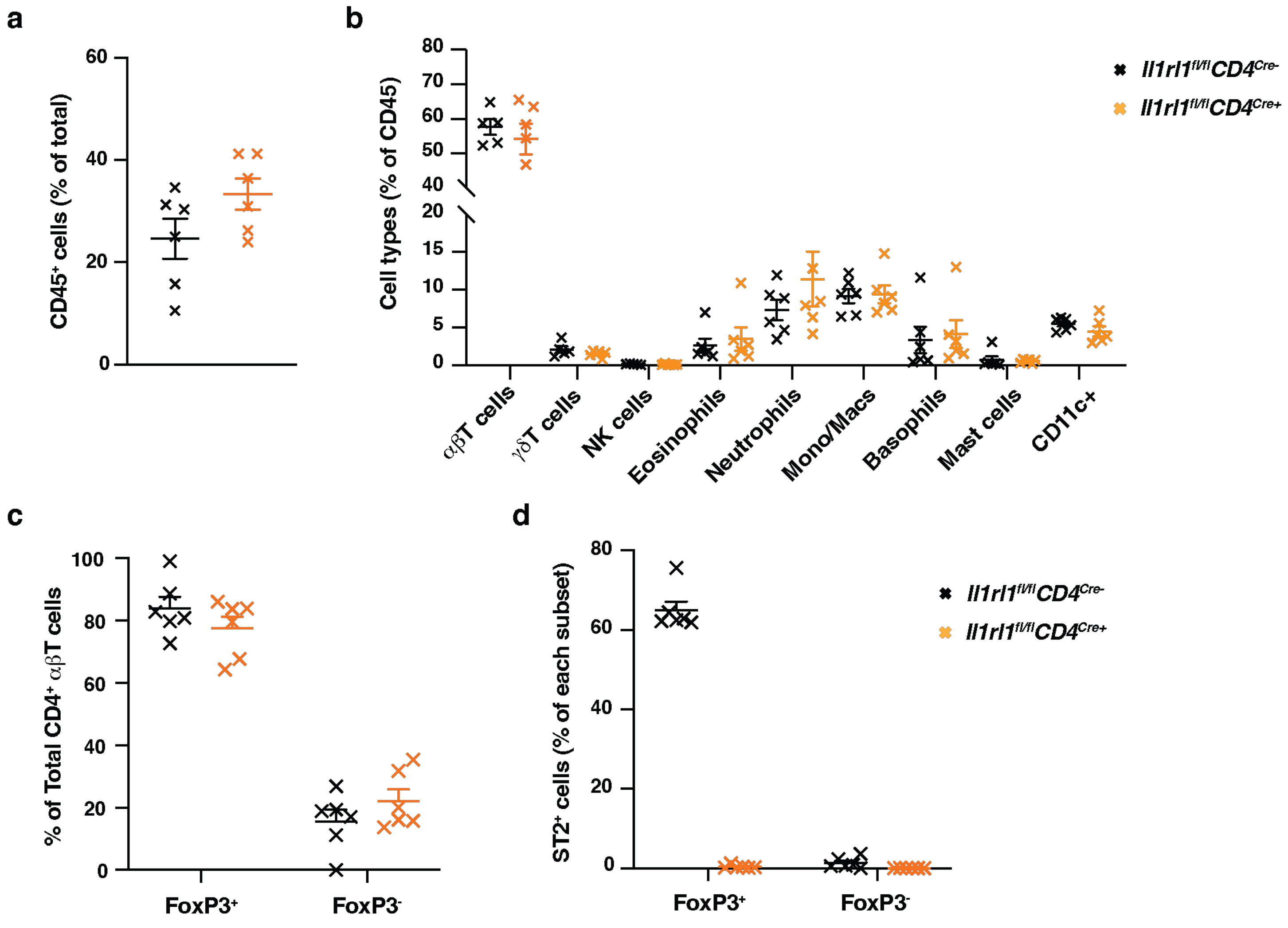

Supplementary figure 8 | The abundance or relative distribution of the tumour leukocyte infiltrate does not differ in the absence of ST2 on CD4 T cells only

cSCCs was induced by DMBA-TPA carcinogenesis on *Il1rl1*$^{fl/fl}$*CD4*$^{Cre-}$ (ST2 WT littermate controls) and *Il1rl1*$^{fl/fl}$*CD4*$^{Cre+}$ mice. Tumours were collected at 18 weeks and the leukocyte infiltrate analysed by flow cytometry. (a) Proportion of total CD45^{+} leukocytes in the tumour tissue as % of live cells in the tumour. (b) Major lymphoid and myeloid subpopulations in the tumour tissue presented as the proportion of total CD45^{+} leukocytes. (c) Tumour CD4^{+} αβ T cells divided into FoxP3^{+} Treg and FoxP3^{-} effector cells as a proportion of total CD4^{+} αβ T cells. (d) ST2 expression on FoxP3^{+} Treg and FoxP3^{-} effector cells as a proportion of each T cell subset. n=6 for each genotype.

Supplementary figure 9

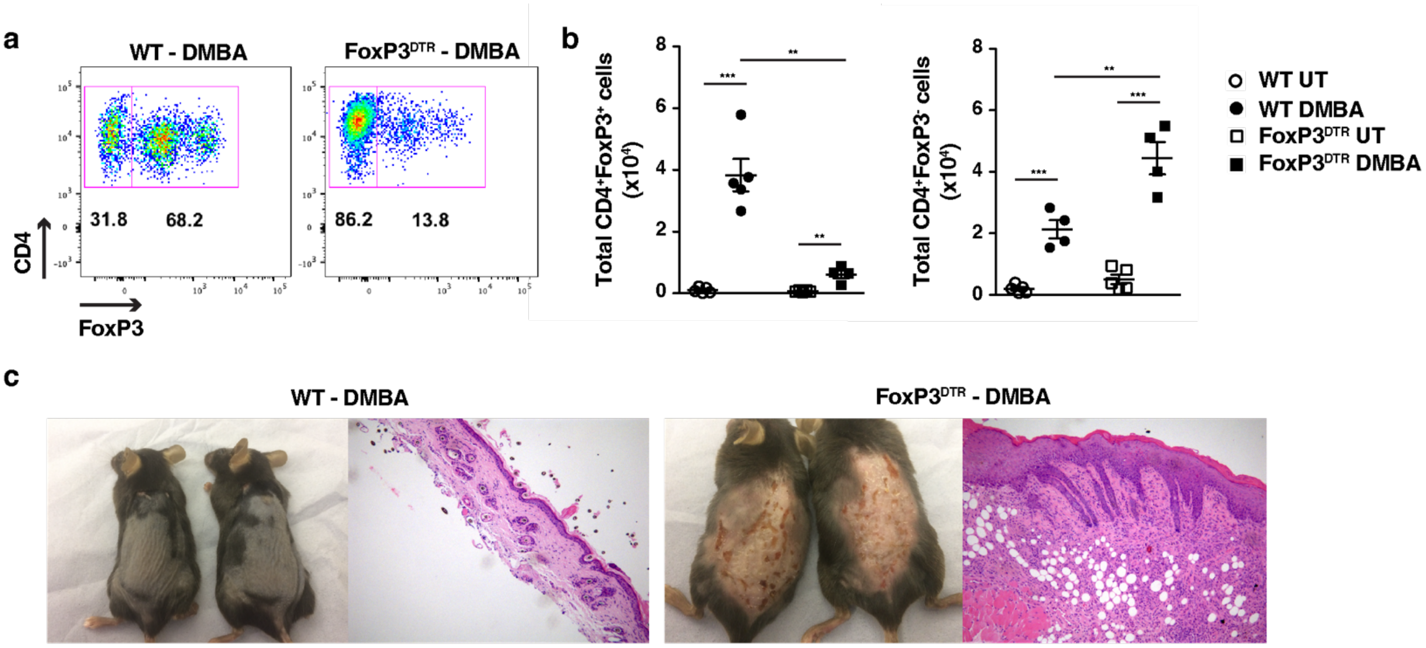

Supplementary figure 9 | Depletion of FoxP3^{+} Treg during topical carcinogen exposure results in severe skin damage

WT and FoxP3^{DTR} mice were injected i.p with 0.25µg Diphtheria toxin and one week later exposed topically to DMBA on the dorsal ear skin (2x DMBA, 3 days apart) or left UT. (a) Representative dotplots showing CD4^{+} Foxp3^{+} Treg in the skin as assessed by flow cytometry 7 days after DMBA exposure and (b) data quantified as total numbers of CD4^{+} Foxp3^{+} Treg and CD4^{+} Foxp3^{-} effector cells in the skin (UT WT controls, open circles (n=5); DMBA treated WT, closed circles (n=4); UT Foxp3^{DTR}, open squares (n=5); DMBA treated Foxp3^{DTR}, closed squares (n=4)). Data shown as means ± SEM. Statistics by two=tailed Student's t-test; ** p<0.01, ***p<0.001. (c) WT and Foxp3^{DTR} mice underwent DMBA-TPA carcinogenesis and were injected with 0.25mg Diphtheria toxin once weekly. The skin was analysed at week 6 and the images show gross appearance of the skin pathology and H+E histology and are representative of n=6. Data are representative of two independent experiments with similar results.

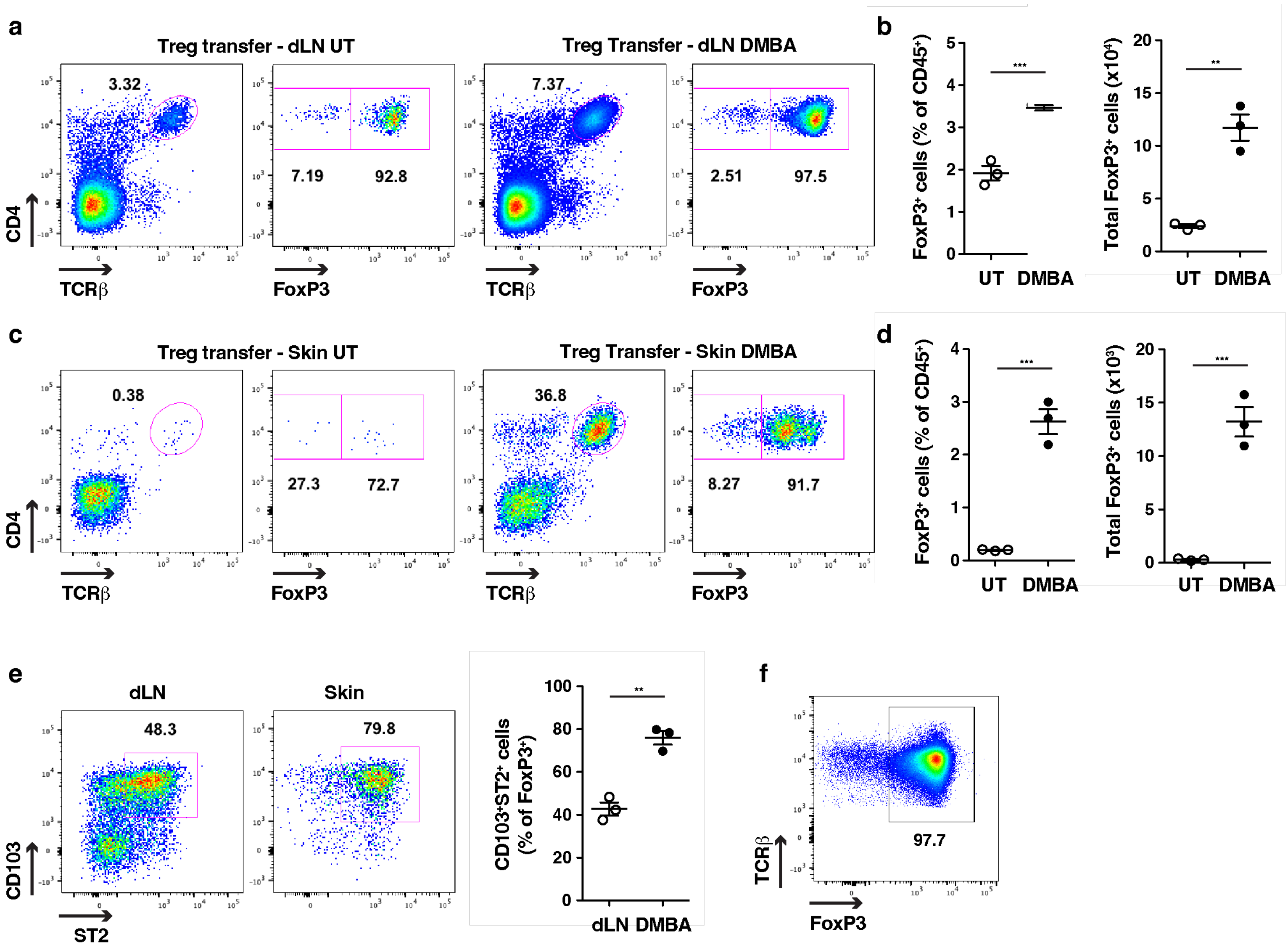

Supplementary figure 10 | Adoptive transfer of CD4$^+$FoxP3$^+$ cells reconstitute Treg in αβ T cell-deficient mice

CD3$^+$CD4$^+$CD25^{hi} cells were purified using Miltenyi MACS kits from spleen and LNs of WT mice. 5x10^5 cells were injected i.v. into αβ T cell deficient mice (*Tcrb*$^{-/-}$). 24h after Treg transfer, the mice were exposed to DMBA topically 2x three days apart (n=3) or left UT (n=3) and the skin dLNs and skin were analysed 7 days later by flow cytometry. (a,c) Representative dotplots showing the presence of TCRb+CD4+FoxP3+ Treg in (a) skin dLNs and (c) skin of *Tcrb*$^{-/-}$ mice following adoptive transfer and with UT or DMBA-treated skin. (b,d) Enumeration of FoxP3$^+$ Treg as a proportion (%) of the total CD45$^+$ population and as total FoxP3$^+$ cells in (b) skin dLN and (c) skin of UT and DMBA-treated mice. (e) Representative dotplots illustrating expression of CD103 and ST2 on Foxp3$^+$ cells in the skin dLN and in the skin following topical DMBA exposure and enumeration of CD103$^+$ST2$^+$ Treg as a proportion (%) of total Foxp3$^+$ Treg. (f) Exemplar dotplot of Treg purity prior to adoptive transfer. Data are expressed as means ± SEM. Statistics by two-tailed Student's t-test; ** $p<0.01$, ***$p<0.001$.

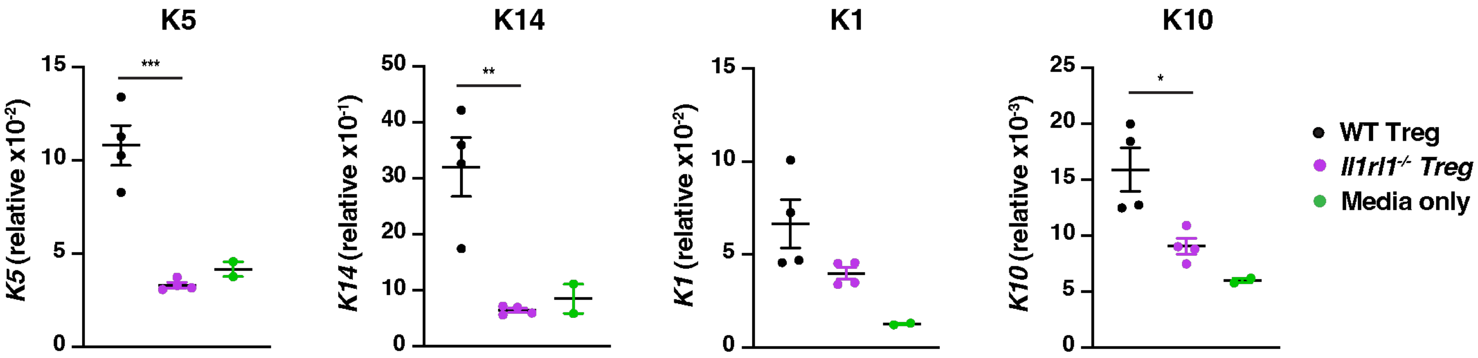

Supplementary figure 11 | IL-33 endows Treg with the ability to induce skin EC differentiation *in vitro*
Primary neonatal WT skin ECs (keratinocytes) were grown *in vitro* to 70% confluency and then supplemented with media from WT or *Il1rl1*$^{-/-}$ Treg stimulated with IL-33, or with media alone (n=4 for Treg groups; n=2 for media only). ECs were analysed by qRT-PCR for expression of transcripts indicating EC differentiation (K5, K14, K1 and K10) relative to the control gene cyclophilin. Data are presented as mean ± SEM and statistics by unpaired t test; * $p<0.05$, ** $p<0.01$ and ***$p<0.001$.

Supplementary Table I | Genes contained in each gprofiler pathway

Pathways	Genes
**Keratinization**	KRT17,DSC2,GM11555,KRT40,GM9507,GM10100,GM10272,KRTAP5-4,GM19668,KRTAP3-2,KRT18,KRT31,GM10024,KRTAP11-1,KRTAP6-3,KRT83,KRT33B,GM10142,KRTAP27-1,KRTAP19-3,KRTAP16-3,KRTAP19-4,KRTAP19-2
**Epidermis development**	SOX9,KRT17,FOXN1,SPRR1A,DLX3,HOXC13,KCNQ1,PDZD7,MSX2,GM1966,KRTAP11-1,KRTAP6-3,KRTAP27-1,KRTAP19-1
**Skin development**	SOX9,KRT17,FOXN1,SPRR1A,DLX3,HOXC13,ATP8A2,MSX2,GM19668,KRTAP11-1,KRTAP6-3,KRTAP27-1,KRTAP19-1
**Hair follicle development**	SOX9,KRT17,FOXN1,DLX3,HOXC13,MSX2
**Skin epidermis development**	SOX9,KRT17,FOXN1,DLX3,HOXC13,MSX2
**Epidermal cell differentiation**	KRT17,FOXN1,SPRR1A,DLX3,KCNQ1,PDZD7,MSX2,GM19668,KRTAP11-1,KRTAP27-1
**Hair cycle process**	SOX9,KRT17,FOXN1,DLX3,HOXC13,MSX2
**Developmental Biology**	KRT17,DOK4,DAB1,EPHB1,DSC2,GM11555,KRT40,GM9507,GM10100,GM10272,KRTAP5-4,GM19668,KRTAP3-2,KRT18,KRT31,GM10024,KRTAP11-1,KRTAP6-3,KRT83,KRT33B,GM10142,KRTAP27-1,KRTAP19-3,KRTAP16-3,KRTAP19-4,KRTAP19-2
**Retinol metabolic process**	CYP1B1,BCO1,PLB1,LRAT,ALDH1A2
**Molting cycle**	SOX9,KRT17,FOXN1,DLX3,HOXC13,MSX2

Supplementary Table II | Genes contained in each gprofiler pathway

Pathways	Genes
**Negative regulation of immune system process**	PDCD1LG2,UBASH3A,PDCD1,GIMAP3,ALOX15,CD300LF,OAS3,MMP12,SLFN1,CD300A,KLRD1,CCL12,FGR,KLRK1,LILR4B,CCR2,PTPRC,BTK,CLEC12B,C1QC,CST7,LPXN,CD86,FCER1G,BTNL2,OAS1A,TNFRSF14,STAP1,CD84,CD74,DHX58,NCKAP1L,TSPAN32,TYROBP,IL1RL1,H2-AB1,H2-AA,PLCL2,IFI204,LST1,CCR1,SPN,LRRC17,TBC1D10C,BST2,SPI1,SAMSN1,HAVCR2,PARP14,LAPTM5,CCL2,CD37,IFI202B,GPR68,IL7R,USP18,IRF1,SERPINB9,MILR1,TNFAIP6,TNFAIP8L2,CD274,MDK,EMILIN1,CNR2,LYN,INPP5D,FGL2,SAMHD1
**Positive regulation of cytokine production**	CHIL3,CCL5,GIMAP3,C3,OAS3,MMP12,CLEC9A,CD300C2,FGR,CCR5,KLRK1,CD2,CLEC7A,OAS2,TICAM2,TLR7,GBP5,CCR2,CD40,C3AR1,SLC11A1,CLEC4N,PTPRC,TLR8,FCNA,PTAFR,CYBB,LTB,TLR9,AIM2,CD6,FCER1G,BTNL2,OAS1A,TNFRSF14,AIF1,CD74,DHX58,SASH3,TYROBP,IL1RL1,IFI204,SPN,CCR7,CSF1R,SCIMP,TWIST1,HAVCR2,CARD9,LAPTM5,CCL2,NFAM1,IFI202B,GSDMD,BATF,IL16,ITK,IRF8,H2M3,IRF1,CD3E,FCER1A,CYBA,CD274,MDK,CLEC5A,CCDC88B,CASP4,ADAM8,NLRP1B,PLA2R1,CD36,RFTN1,GPSM3,CD83,LUM,POSTN,STAT1,NFATC4,POU2F2
**Positive regulation of response to external stimulus**	F7,CCL5,KLRC1,GIMAP3,C3,MMP12,KLRD1,CCR5,KLRK1,CLEC7A,ZBP1,CXCL10,GBP5,CCR2,IL18RAP,C3AR1,CLEC4N,CTSS,FCGR1,IRGM1,TLR8,FCNA,BTK,RAC2,CCL7,IGTP,TLR9,AIM2,CXCR3,FCER1G,STAP1,AIF1,CD74,DHX58,NCKAP1L,TYROBP,IL1RL1,VAV1,IFI204,CCR1,LY86,CCR7,CSF1R,SCIMP,SPI1,HAVCR2,CARD9,CCL2,IFI202B,CD180,ALOX5AP,IL16,H2-M3,FCER1A,CYBA,MDK,EMILIN1,PIK3CG,CASP4,EMILIN2,ADAM8,NLRP1B,PDGFRB,PARP9,SCARF1
**Regulation of leukocyte proliferation**	CCL5,PDCD1LG2,SLFN1,RASAL3,CD300A,IKZF3,CCL12,ZAP70,LILR4B,CCR2,CD40,PTPRC,ITGAL,BTK,RAC2,TLR9,CD6,CD86,CD209A,BTNL2,CORO1A,TNFRSF14,AIF1,CD74,NCKAP1L,SASH3,TYROBP,H2-AB1,H2-AA,LST1,SPN,CCR7,CSF1R,CD4,VCAM1,HAVCR2,LAPTM5,IRF1,CD3E,CD274,LYN,GPR183,INPP5D,HHEX
**Regulation of immune effector process**	KLRC1,GIMAP3,C3,CD300A,KLRD1,FGR,KLRK1,CLEC7A,LILR4B,CCR2,IL18RAP,C6,CD40,TNFSF13,PTPRC,FCGR1,BTK,PTAFR,RAC2,TLR9,CLEC12B,CD86,FCER1G,TNFRSF14,STAP1,CD84,WAS,CD74,DHX58,PIK3R6,NCKAP1L,SASH3,TYROBP,VAV1,SPN,CCR7,SCIMP,BST2,SPI1,HAVCR2,LAPTM5,CCL2,CD37,IL7R,H2-M3,IRF1,SERPINB9,FCER1A,LYN,NCF1,FGL2
**Regulation of mononuclear cell proliferation**	CCL5,PDCD1LG2,SLFN1,RASAL3,CD300A,IKZF3,ZAP70,LILR4B,CCR2,CD40, PTPRC,ITGAL,BTK,RAC2,TLR9,CD6,CD86,CD209A,BTNL2,CORO1A,TNFRSF14,AIF1,CD74, NCKAP1L,SASH3,TYROBP,H2-AB1,H2-AA,LST1,SPN,CCR7,CSF1R, CD4,VCAM1,HAVCR2,LAPTM5,IRF1,CD3E,CD274,LYN,GPR183,INPP5D
**Cytokine-mediated signaling pathway**	CCL17,CCL5,CD300LF,CXCR6,OAS3,MMP12,OASL2,CCL12,CXCL9,CCR5, CCL9,ZBP1,OAS2,CXCL10,IL21R,TICAM2,CCR2,IIGP1,IL18RAP,NAIP6,CCL8, PTPRC,IRGM1,CCL7,IGTP,AIM2,CXCR3,FCER1G,OAS1A,STAP1,PF4,IL10RA, CCL22,CD74,IL1RL1,IL2RG,CCR1,CCR7,CSF1R,CD4,SPI1,PARP14,LAPTM5, CCL2,IL7,CCL6,USP18,IRF1,NAIP2,CCL11,SAMHD1,IL2RB,CASP4,GREM2, TNFRSF1B,EBI3,PARP9,ACKR1,STAT1,IFITM1,GAS6,SOCS1,CSF2RA,CSF1, JAK3,STAT2,FAS
**Regulation of response to biotic stimulus**	CCL5,KLRC1,GIMAP3,C3,OAS3,MMP12,KLRD1,FGR,KLRK1,CLEC7A,ZBP1, GBP5,IL18RAP,CLEC4N,IRGM1,TLR8,FCNA,IGTP,TLR9,CLEC12B,AIM2, OAS1A,CD74,DHX58,PIK3R6,TSPAN32,TYROBP,VAV1,IFI204,CCR1,LY86, SPN,SCIMP,SPI1,HAVCR2,PARP14,CARD9,CD37,IFI202B,CD180,USP18,H2-M3,IRF1,SERPINB9,CYBA,CD274,EMILIN1,NCF1,FGL2,SAMHD1
**Mononuclear cell proliferation**	CCL5,PDCD1LG2,SLFN1,RASAL3,CD300A,IKZF3,ZAP70,LILR4B,CCR2,CD40, SLC11A1,PTPRC,ITGAL,BTK,RAC2,TLR9,CD6,CD86,CD209A,DOCK2,BTNL2, CORO1A,TNFRSF14,AIF1,CD74,NCKAP1L,SASH3,TYROBP,H2-AB1,H2-AA, PLCL2,LST1,SPN,CCR7,CSF1R,CD4,VCAM1,HAVCR2,LAPTM5,IL7R,CD180, IRF1,CD3E,CD274,LYN,GPR183,INPP5D
**Leukocyte proliferation**	CCL5,PDCD1LG2,SLFN1,RASAL3,CD300A,IKZF3,CCL12,ZAP70,LILR4B,CCR2, CD40,SLC11A1,PTPRC,ITGAL,BTK,RAC2,TLR9,CD6,CD86,CD209A,DOCK2, BTNL2,CORO1A,TNFRSF14,AIF1,CD74,NCKAP1L,SASH3,TYROBP,H2-AB1, H2AA,PLCL2,LST1,SPN,CCR7,CSF1R,CD4,VCAM1,HAVCR2,LAPTM5,IL7R,CD180,IRF1,CD3E,CD274,LYN,GPR183,INPP5D,HHEX
